# Quantitative chemical mapping of plagioclase as a tool for the interpretation of volcanic stratigraphy: an example from Saint Kitts, Lesser Antilles

**DOI:** 10.1007/s00445-021-01476-x

**Published:** 2021-07-16

**Authors:** Oliver Higgins, Tom Sheldrake, Luca Caricchi

**Affiliations:** Department of Earth Sciences, University of Geneva, rue des Maraîchers 13, 1205 Geneva, Switzerland

**Keywords:** Magma, Image segmentation, Zoning, Crystal population, Anorthite

## Abstract

Establishing a quantitative link between magmatic processes occurring at depth and volcanic eruption dynamics is essential to forecast the future behaviour of volcanoes, and to correctly interpret monitoring signals at active centres. Chemical zoning in minerals, which captures successive events or states within a magmatic system, can be exploited for such a purpose. However, to develop a quantitative understanding of magmatic systems requires an unbiased, reproducible method for characterising zoned crystals. We use image segmentation on thin section scale chemical maps to segment textural zones in plagioclase phenocrysts. These zones are then correlated throughout a stratigraphic sequence from Saint Kitts (Lesser Antilles), composed of a basal pyroclastic flow deposit and a series of fall deposits. Both segmented phenocrysts and unsegmented matrix plagioclase are chemically decoupled from whole rock geochemical trends, with the latter showing a systematic temporal progression towards less chemically evolved magma (more anorthitic plagioclase). By working on a stratigraphic sequence, it is possible to track the chemical and textural complexity of segmented plagioclase in time, in this case on the order of millennia. In doing so, we find a relationship between the number of crystal populations, deposit thickness and time. Thicker deposits contain a larger number of crystal populations, alongside an overall reduction in this number towards the top of the deposit. Our approach provides quantitative textural parameters for volcanic and plutonic rocks, including the ability to measure the amount of crystal fracturing. In combination with mineral chemistry, these parameters can strengthen the link between petrology and volcanology, paving the way towards a deeper understanding of the magmatic processes controlling eruptive dynamics.

**Supplementary Information:**

The online version contains supplementary material available at 10.1007/s00445-021-01476-x.

## Introduction

The interplay between the chemical and physical processes experienced by magma within the crust is intimately linked to the style and frequency of eruptions observed at the surface (Baker and Holland, 1973; Gertisser and Keller, 2003). Volcanic stratigraphy provides a snapshot of these chemical and physical processes, and holds a plethora of opportunities to collect qualitative and quantitative data within a temporal context: changes in mineral chemistry (Sisson and Vallance, 2009) and modal mineralogy (Luhr and Carmichael, 1982), whole rock geochemical variation (Gertisser and Keller, 2003) and textural quantification in the form of crystal size distributions (Higgins and Roberge, 2003). Magmatic minerals can also be exploited for temporal geochemical studies by fingerprinting the state of a magmatic system as they grow (Ginibre et al., 2007; Wallace and Bergantz, 2002). Together, volcanic stratigraphy and mineral chemistry can be effectively combined to integrate physical volcanology and petrology in a temporal framework (Kahl et al., 2013), improve our understanding of open system processes in volcanic arcs (Humphreys et al., 2006; Viccaro et al., 2010), and further our ability to use correlation of mineral zoning as a tool for geological mapping and tephrostratigraphy (Wiebe, 1968).

Plagioclase provides a robust crystal record due to its near-ubiquitous occurrence in magmas, and compositional sensitivity to temperature, melt composition and melt water content (Sisson and Grove, 1993). Minerals record the temporal evolution of their conditions of growth as chemical and textural zoning (Davidson et al., 2007), which can exhibit a wide variety of styles including normal, reverse, oscillatory and patchy zonation (Ginibre et al., 2007; Viccaro et al., 2010). Quantifying the chemical and textural variability of plagioclase is challenging due to “petrological cannibalism” whereby injected magma may scavenge thermally, chemically and spatially disparate crystals from the plutonic subsystem that underlies a volcano and amalgamate them into a final erupted product (Cashman and Blundy, 2013; Davidson et al., 2007; Reubi and Blundy, 2008). As a result, individual samples may contain numerous crystal populations that are chemically and texturally distinct from each other, as well as single crystals that record several chemo-physical magmatic states in their zoning patterns (Kent et al., 2010; Wallace and Bergantz, 2005). When considering an entire eruptive sequence, crystal populations may appear and disappear in time between erupted products, reflecting recurring magmatic processes. Arc volcanoes in particular (e.g. Saint Kitts in the Lesser Antilles), generally erupt magmas with highly composite whole rock and crystal textures, notably the case for plagioclase (Humphreys et al., 2013; Reubi and Blundy, 2009). This is reflective of the often-complex nature of volcanic plumbing systems where magma may be stored and evolve at different depths, (Melekhova et al., 2017) and experiences mixing (Kent et al., 2010) and/or mingling (Howe et al., 2015).

Past attempts to quantify plagioclase textural and chemical variability can be divided into crystal-scale and sample-scale methods. Crystal-scale methods employ multiple crystal transects, using a variety of mathematical approaches to identify discrete populations (Caricchi et al., 2020; Probst et al., 2018; Wallace and Bergantz, 2005, 2002). Transects are commonly acquired on selected, large (> 300 µm) crystals using an electron probe micro-analyser (EPMA). These analyses are highly accurate (≤ 1% relative error) and rapidly acquired (~ 3 min per point). However, recording the full range of 2D zoning patterns and their relative importance, i.e. their areal extent, using transects alone is clearly difficult owing to stereological constraints and under sampling (Probst et al., 2018). Quantifying sample-scale variation has more commonly been approached using back-scattered electron (BSE) maps of thin sections (Cheng et al., 2017; Cheng and Costa, 2019; Humphreys et al., 2013; Zeng et al., 2018), for example by calibrating BSE greyscale images with the punctual determination of anorthite content (Ginibre et al., 2002). Sheldrake and Higgins (2021) presented a method using image segmentation of X-ray maps to classify texturally constrained zones of similar chemical composition. This allows single-plagioclase crystals to be split into multiple zones, which can then be correlated within and between samples.

This study centres on a well-exposed volcano-stratigraphic section on an island arc volcano (Saint Kitts, Lesser Antilles) that represents a millennial timescale of eruptive history. Given its ubiquity throughout the stratigraphy, we use plagioclase to provide us with a “crystals-eye view” of the magmatic plumbing system, using a series of quantified chemical maps to interrogate mineral chemistry. Our results show that the systematic investigation of plagioclase chemistry can reveal temporal trends not evident in whole rock geochemistry alone. Using the method of Sheldrake and Higgins (2021), we quantify the evolution of textural complexity of plagioclase in time. Using this method, we also measure the degree of crystal fracturing with a new textural parameter (fracture index), which has the potential to provide quantitative information on magma decompression rates (Benage et al., 2021; Miwa and Geshi, 2012; Taddeucci et al., 2021).

## Geological setting

**Fig. 1:**
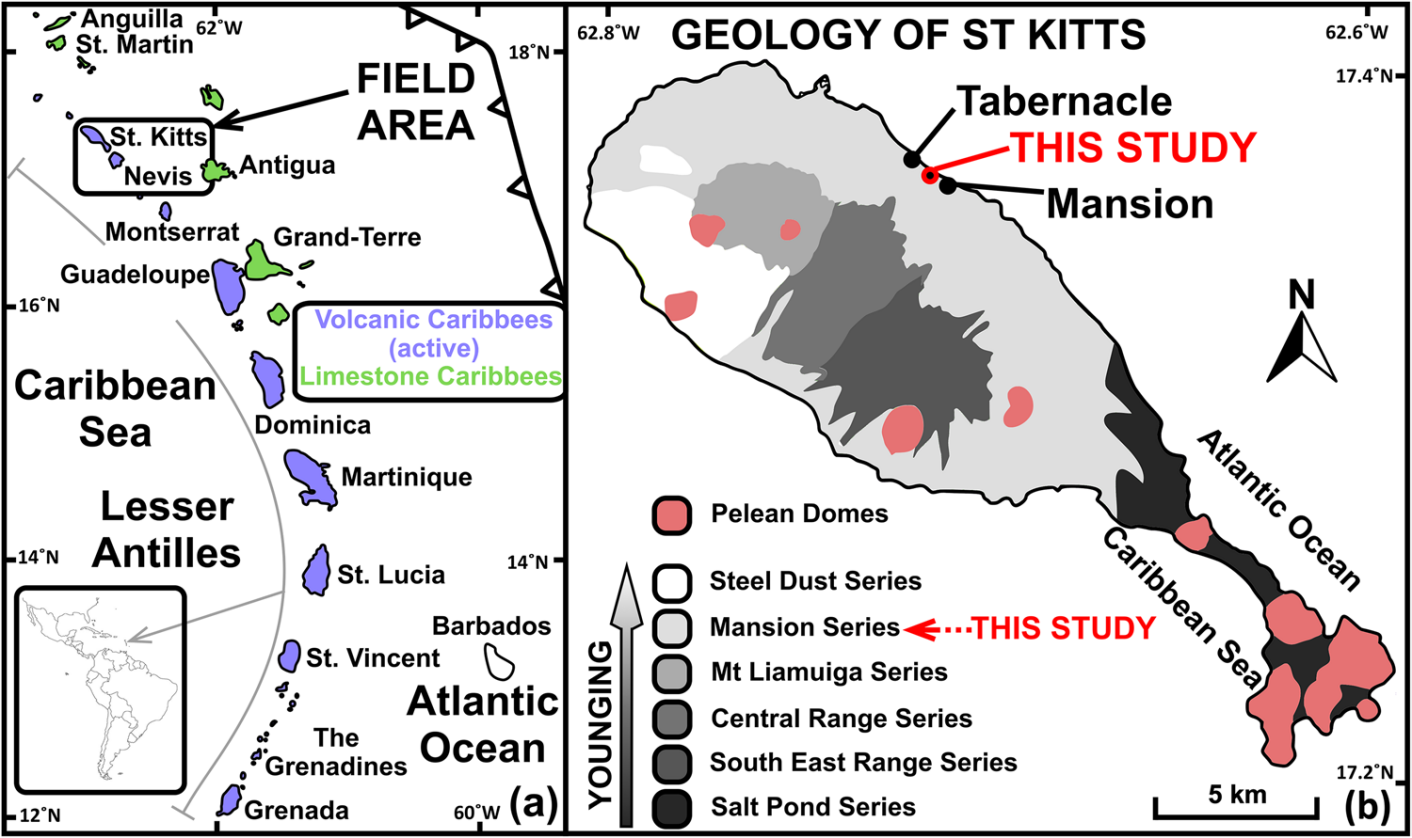
**a** Map of the Lesser Antilles island arc modified after Toothill et al. (2007). The arc is divided into the active Volcanic Caribbees (west) and inactive Limestone Caribbees (east). Saint Kitts, the field area for this study, is located at the northern tip of the Volcanic Caribbees. **b** Geological map of Saint Kitts, Lesser Antilles, modified after Geologist and Martin-Kaye (1959). The island consists of four volcanic centres which young towards the NW. Peléan Style volcanic domes of various ages outcrop across the island (e.g. Baker, 1968). Study locality is a sea cliff showing a well-exposed pyroclastic sequence between the villages of Mansion and Tabernacle

The island of Saint Kitts is located in the north of the Lesser Antilles island arc, the surface manifestation of the slow (2–4 cm/year; Wadge and Shepherd, 1984), westward subduction of the North American plate beneath the Caribbean Plate (Fig. 1a). The Lesser Antilles is renowned for its chemical diversity both along the arc (Macdonald et al., 2000) and within single islands (e.g. Stamper et al., 2014), with the whole rock compositions of volcanic and plutonic rocks spanning almost the entire global arc array (Melekhova et al., 2019). Eruptions are predominantly Plinian and sub-Plinian with interspersed periods of lava dome growth, as seen most recently on Saint Vincent (27^th^ December 2020–present) but also noted on Saint Kitts, Montserrat, Dominica and Martinique (Baker, 1968; Loughlin et al., 2010).

Saint Kitts is host to four main volcanic centres (Fig. 1b). Volcanic activity has migrated from the Salt Pond Peninsula centre in the south (2.3 Ma; Baker, 1968) to the active Mt Liamuiga centre in the north (Baker, 1968). Parasitic Peléan domes outcrop across the island around each of the four main centres. There are no dacites on Saint Kitts, and the only example of rhyolite crops out on Scotch Bonnet, a promontory at the south-eastern end of the Salt Pond Peninsula. The central and south-eastern parts of the island are composed mostly of pyroxene-andesite lava flows, domes and agglomerates (Baker and Holland, 1973). Saint Kitts provides a useful end member for the northern Antilles with respect to its trace element variation and low K_2_O content in the erupted products (Macdonald et al., 2000).

Mt Liamuiga is the active stratovolcano in the north of the island. Activity initiated ~ 42 ka (Roobol et al., 1981), with no confirmed reports of eruptions after its European settlement in 1624 despite continued fumarolic activity. Eruption products are typically basaltic and basaltic-andesite lava flows, pyroclastic flows and fall deposits (this study; Toothill et al., 2007). The volcanic units of Mt Liamuiga are grouped into the Mansion Series, first described by Baker (1968), which is one of the best-preserved volcanic sequences of any Lesser Antilles island. It derives its name from the type locality in a ravine below Mansion village (Fig. 1b), with a new type locality proposed at Phillips Gut after the covering of lower strata by a local rubbish tip (Roobol et al., 1985). The original subgroupings of the Mansion Series from Baker (1968) were revised by Roobol et al. (1981) to consist of 6 main groups (units A–F). The oldest deposits, the focus of this study, are the Lower Green Lapilli (A) and Upper Green Lapilli (C) which are separated by the Cinder Unit (B). The Green Lapilli layers consist primarily of grey-green, angular, aphyric, micro-vesicular lapilli of andesitic composition, a rock type not noted elsewhere in the Lesser Antilles. They have been interpreted by Baker (1980) as fragments of volcanic bombs due to their faceted, rhomboidal form, and by Roobol et al., (1985) as phreatomagmatic deposits due to their lack of vesicles and angularity. The eruptive sequence resumes at 4270 BP ± 140 until 2070 ± 150 (Baker, 1985) with units D–F. They comprise of interbedded ash, pumice fall deposits and pyroclastic flow deposits, along with intercalations of the Steel Dust Series fall deposits on the western flanks of Mt Liamuiga.

## Methods

### Fieldwork

**Fig. 2:**
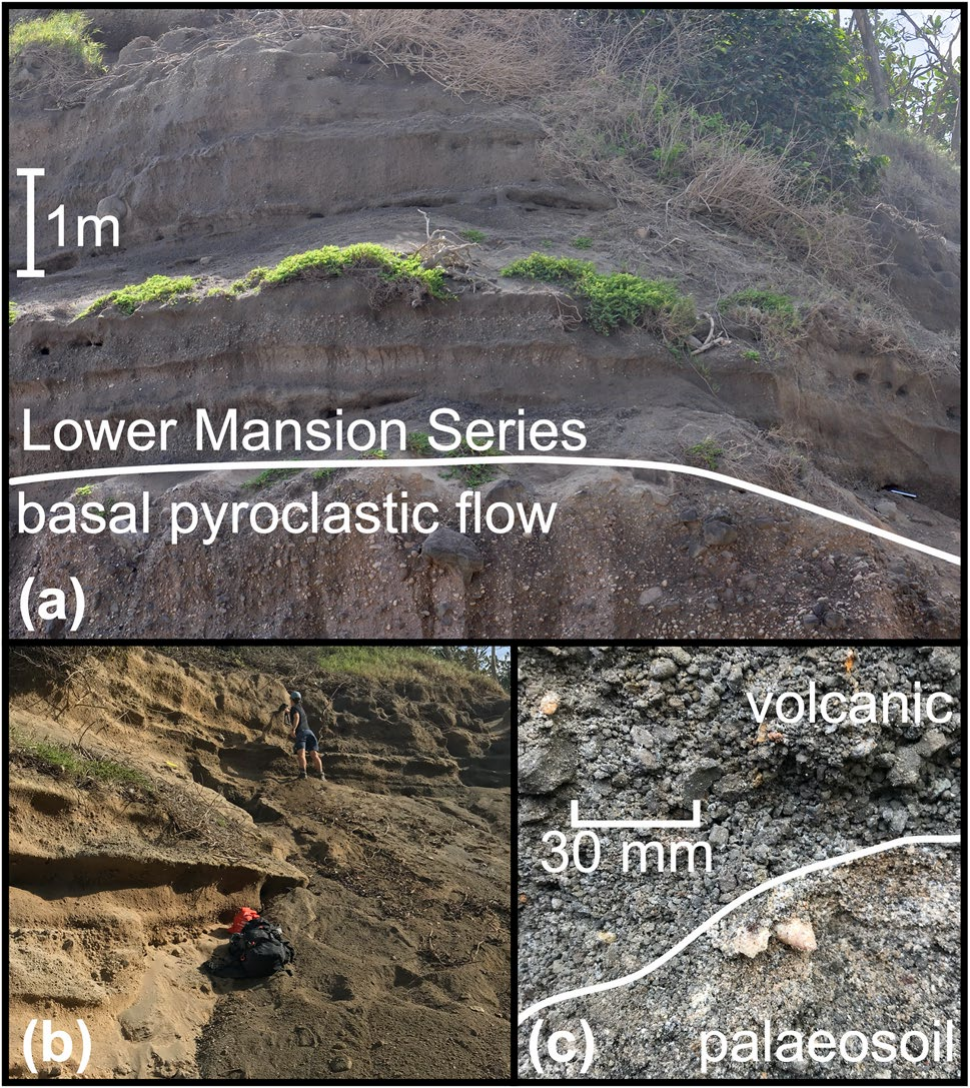
**a** Stratigraphic sequence that is the focus of this study. A basal pyroclastic flow deposit separates the Lower Mansion Series (units A–C according to Roobol et al., 1981). Strata are subhorizontal and show no signs of reworking. **b** Material was excavated to retrieve a fresh surface for sampling and field measurements. **c** A land snail (genus: *Cerion*) inside a palaeosoil layer

Samples were collected from a 6.8-m-thick, well-exposed stratigraphic section on the east coast of Saint Kitts (17.38725, − 62.76276 [WGS84]; halfway between the villages of Mansion and Tabernacle; Fig. 2). This section encapsulates the “Pre-Mansion Series pyroclastic deposits” (> 43,000 BP) and Mansion Series units A–C (> 41,420 to > 41,730 BP) which have been dated using ^14^C (Harkness et al., 1994; Roobol et al., 1981). The outcrop was first cleaned with a shovel to reach a fresh surface. Thicknesses between units were measured and representative samples of juvenile material collected for chemical analysis. Juvenile material was considered as pumice, mafic scoria or volcanic ash. Palaeosoils were distinguishable by colour, texture, fragments of disaggregated pumiceous material and the presence of Cerion (a genus of tropical land snail). Beds were sampled at clear changes in deposit form or macroscopic mineralogy in order to capture the full variability of the sequence. Basic field observations are summarised in Online Resource 1, Table S1.

### Sample preparation

Polished, 30-µm thin sections of juvenile material were made for chemical and petrographical analysis by Jean-Marie Boccard at the University of Geneva. The exception was SK391 which was mounted on a polished circular mount. Macroscale textures include pumice with elongate vesicles (e.g. SK408), microvesicular pumices with sparse phenocrysts (e.g. SK386B), and mafic scoria with abundant phenocrysts of plagioclase and crystal clots (SK392). Scans of thin sections in plane polarised light can be found in Online Resource 2, Fig. S1.

### Whole rock major and trace elements

Selected samples were cleaned and dried in an oven at 60 °C for 24 h. The dried samples were then crushed, sieved to < 500 µm and milled to a fine, homogeneous powder using an agate mill. Glass beads were made from the resulting powder using a PANalytical EAGON-2 fusion machine on a pre-set silicate programme at the University of Geneva. Major element analysis was performed on the glass beads by X-ray fluorescence (XRF) using a PANalytical AXIOS MAX with a rhodium anode tube at 4 W at the University of Lausanne. DA-12, NIM-N and NIM-G standards were used for quality control. A suite of trace elements was measured on the glass beads by laser ablation inductively coupled plasma mass spectrometry (LA-ICP-MS) at the University of Lausanne using a Quadrupole Agilent 7700 spectrometer interfaced to a GeoLas 200 M 193-nm excimer ablation system. The NIST SRM 612 external standard was measured at the beginning and end of every four unknowns. Data were reduced using the SILLS data reduction software (Guillong et al., 2008) with SiO_2_ (wt%) from XRF analysis used as the internal standard. Major element whole rock measurements and selected trace elements for the samples used in this study are found in Online Resource 3, Table S2.

### EPMA

In situ mineral analyses of plagioclase were performed using a JEOL 8200 Superprobe at the University of Geneva and a JEOL JXA-8530F at the University of Lausanne. Both microprobes were equipped with a five-channel wavelength-dispersive spectroscope system (WDS) and were operated at an accelerating voltage of 15 keV, a beam current of 15 nA and a beam diameter of 5 µm. Quantitative analyses were made using a variety of internal standards (orthoclase [Si, K], andalusite [Al], albite [Na], forsterite [Mg], fayalite [Fe], wollastonite [Ca], Mn-Ti oxide [Mn, Ti], Cr oxide [Cr]). Mineral analyses were targeted to span the full variability of plagioclase zoning in a given thin section, typically using transects. WDS maps were measured on a representative part of 9 selected thin sections using a 20 µm pixel size to map a total area of ≥ 100 mm^2^ (≥ 250,000 pixels) with an analysis time of ~ 21 h per sample (10 elements, 2 passes). Analysis conditions were 15 keV, 100 nA, 150 ms dwell time and a 5-µm beam. All plagioclase EPMA data are presented in Online Resource 4, Table S3.

### Textural segmentation

**Fig. 3:**
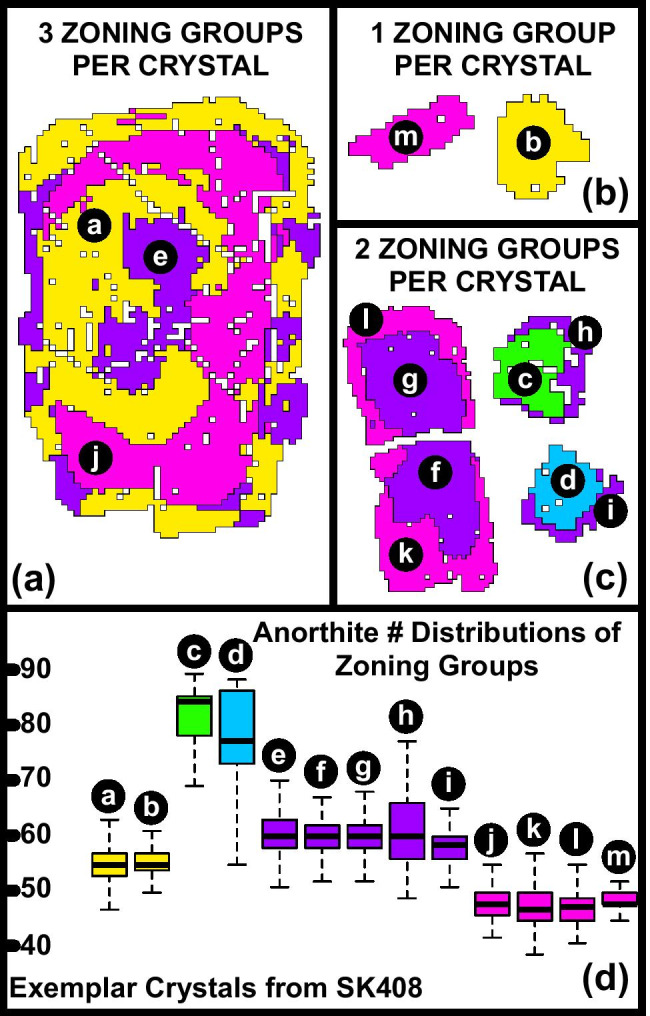
Example of image segmentation of plagioclase zoning inside exemplar crystals from sample SK408. White areas within a crystal represent mixed pixels (e.g. containing melt inclusions, cracks, etc.). In this example, segmentation splits plagioclase crystals in up to three distinct spatial-chemical zones (**a**–**c**). Zoning patterns from segmentation match well with observations from Ca and Na zoning in chemical maps. The An# distributions of all segmented zones from all crystals within the stratigraphy are compared using hierarchical clustering to produce zoning groups (colours in Fig. 3) in which An# distributions are comparable (**d**). Crystals are not to scale. Letters are to be used to match zoning domains to each individual barplot in **d**

Phase separation was performed using a finite mixture model on the EPMA count maps of all 10 measured elements (Sheldrake and Higgins 2021). This produced a phase map of all silicate and oxide minerals, as well as the matrix of microlites and silicate glass (Online Resource 5, Fig. S2). Plagioclase was then isolated from the phase map for textural segmentation. For each plagioclase crystal in turn, spatially constrained regions of pixels with similar chemical composition (superpixels) were identified (Sheldrake and Higgins 2021). Superpixels were initiated around a grid of regularly spaced central pixels (centroid). The algorithm then iteratively searches in a grid around each superpixel centroid, calculating a spatial-chemical distance between each pixel in the grid and the respective centroid. The spatial-chemical distance is a weighted function based on the spatial difference (i.e. in X and Y coordinates) and chemical difference (i.e. in normalised counts of Na and Ca). We used the default weights presented in Sheldrake and Higgins (2021). If the new spatial-chemical distance is smaller than the previous iteration, the given pixel is assigned into that superpixel. Superpixels within the crystal were then compared using an affinity propagation (AP) algorithm, which generates a similarity matrix and groups together similar superpixels. These groups of superpixels are spatially and chemically defined, and their shape and distribution match with zoning patterns observed in the crystal (e.g. high anorthite core, low anorthite rim). In order to compare crystals, from the same and different samples, the WDS chemical count maps were calibrated. We used a series of quantitative EPMA points, analysed in the same analytical session, to produce anorthite maps (where anorthite number, An# = [Ca^2+^ / Ca^2+^  + Na^+^  + K^+^] × 100; Online Resource 6, Fig. S3). Uncertainty on anorthite calibrations is typically ≤ 2 mol%, comparable to values from calibrated BSE images (Ginibre et al., 2002). Each segmented zone can then be defined by an anorthite distribution. In this study, segmentation generates crystals that are divided into between 1 and 4 unique chemical zones per crystal (Fig. 3a–c; although this number is not user determined a priori). To correlate the chemical zones, we calculate the median distance between their respective anorthite distributions (Sheldrake and Higgins 2021). Using hierarchical clustering, we classify 13 zoning groups (Online Resource 7, Fig. S4) which are defined as spatial-chemical regions that share comparable anorthite distributions. Crystal populations are then defined as groups of crystals that contain the same combination of zoning group(s) as determined by the segmentation method. The full results of the segmentation (Online Resource 7, Fig. S4) were then qualitatively checked against macroscale textures observable in thin section (Online Resource 2, Fig. S1), quantified An# maps (Online Resource 6, Fig. S3) and selected BSE images (Online Resource 8, Fig. S5).

## Results

### Stratigraphic whole rock geochemistry

**Fig. 4:**
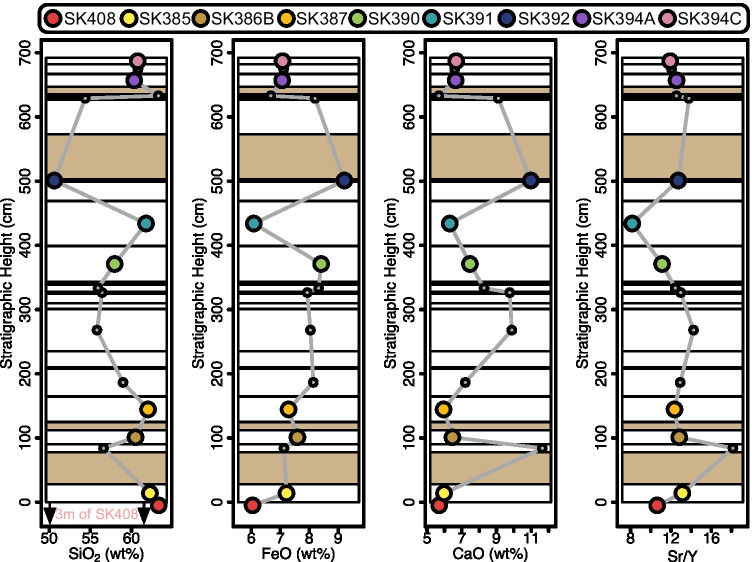
Variation of selected major and trace elements of the Saint Kitts Lower Mansion Series as a function of stratigraphic height (relative age). Samples chosen for quantitative mapping are indicated by large, coloured symbols. Different deposits (see Online Resource 1, Table S1 for details) are separated with horizontal lines. Palaeosoil horizons are shown in light brown

The whole rock SiO_2_ content varies between 50.6 and 63.3 wt% (Fig. 4), spanning much of the total variability on the island (47.5 to 65.4 wt% SiO_2_, excluding the rhyolite at Scotch Bonnet; this study; Baker, 1968; Toothill et al., 2007). In general, no clear pattern in whole rock chemistry emerges in time. SiO_2_ broadly decreases from a maximum at the base (63.6 wt%) to a minimum at 500 cm (50.6 wt%), followed by a resurgence to andesitic values. The silica minimum is followed by a 70-cm-thick palaeosoil layer. This sequence is punctuated by non-systematic oscillations between ~ 56 and ~ 62 wt% SiO_2_. SiO_2_ and CaO variations on Saint Kitts are remarkably well correlated, not only for this sequence but all lava and pyroclastic samples on the island. FeO has a negative correlation with SiO_2_ accordant with Saint Kitts’ classification as tholeiitic–calc-alkaline transitional (Macdonald et al., 2000). There is a notable minimum in iron at 500 cm, consistent with a ~ 4 cm layer of dark, basaltic (Fe-rich) scoria.

### Mineralogy

**Fig. 5:**
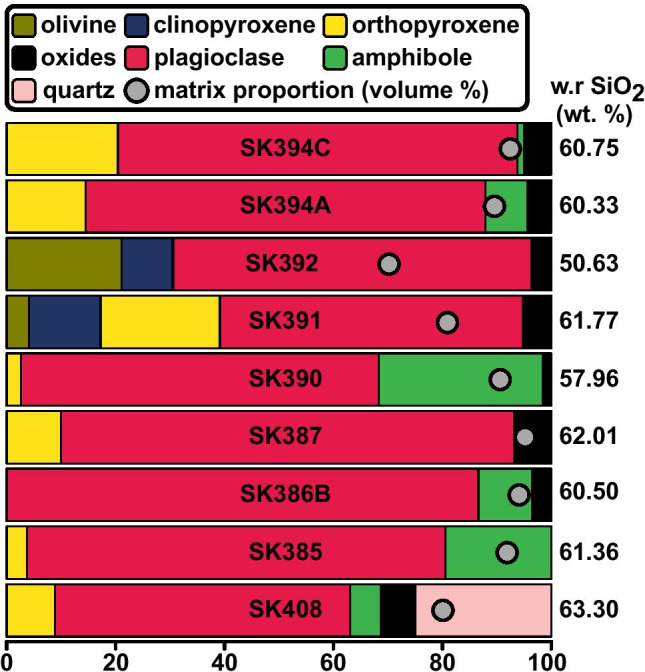
Modal mineralogy of crystals in volume % for all chemically mapped samples in stratigraphic order (oldest at the base). Matrix volume % is shown as grey dots. Whole rock wt% SiO_2_ is reported for each unit on the right of each bar

Mineral phase abundances for all mapped samples (expressed in volume percent) can be seen in Fig. 5. Olivine is present only in SK391 and SK392, forming rounded phenocrysts and monomineralic clots as well as in a reaction relationship with clinopyroxene and orthopyroxene (SK392). In SK392 (a basaltic scoria), olivine has marked normal zoning in some phenocrysts. Plagioclase is a dominant phenocryst throughout (54–83 vol% of phenocryst assemblage), showing no correlation between phase abundance and whole rock SiO_2_. Clinopyroxene only appears in those samples that contain olivine (SK391, SK392), typically associated with orthopyroxene as clots. Orthopyroxene is easily identified by its pale green–pale brown colour in plane polarised light and the coprecipitation of Fe oxides growing as inclusions in the rim and, less commonly, in the core. It is typically unzoned but may exhibit weak oscillatory or reverse zoning (SK408). Where both orthopyroxene and amphibole are present, a higher fraction of orthopyroxene may result in a lower fraction of amphibole (Fig. 5). Amphibole is a common phenocryst phase in the pyroclastic rocks, and in plutonic and cumulate inclusions erupted on Saint Kitts, but is rare to absent in the lava and dome rocks (Baker, 1980; Melekhova et al., 2017; Toothill et al., 2007). Phenocrysts are generally fresh, showing no signs of decompression-induced breakdown or reactions. Oxides are found in all samples excluding SK385. Quartz occurs solely in SK408 as large (< 2.1 mm), rounded crystals, commonly with randomly orientated fractures and embayments. Matrix (including glass and microlites) ranges from 70 to 95 vol%. The matrix proportion does not vary systematically with silica, with the most basaltic sample (SK392) containing a notable abundance of phenocryst phases (70 vol% matrix).

### Plagioclase

**Fig. 6:**
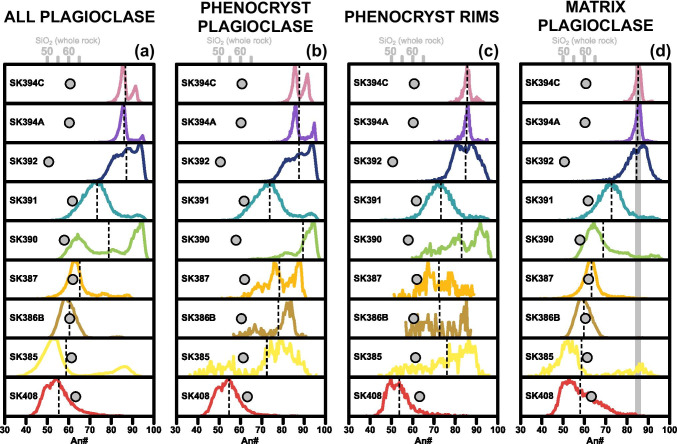
An# distributions of plagioclase for all chemically mapped samples. Panels from left to right are **a** all plagioclase, **b** phenocryst plagioclase that has been included in the textural segmentation (with a crystal area ≥ 81 pixels or 32400 µm^2^), **c** phenocryst rims (outermost pixel of all phenocrysts) and **d** matrix plagioclase (unsegmented plagioclase). Vertical black dashed lines are the mean An# for each distribution. Grey points are whole rock wt% SiO_2_. Phenocrysts generally show bimodal distributions which reflect composite zoning patterns in plagioclase phenocrysts. Matrix plagioclase shows unimodal distributions with slight tails to higher An#, e.g. SK390. Distributions considering all plagioclase show wider distributions that capture features from both segmented and matrix plagioclase. Grey bar in **d** shows An# ~ 85 where the mean of the anorthite distributions for matrix plagioclase becomes invariant at the top of the sequence

Plagioclase varies in its chemistry (expressed as An#) and texture both within and between erupted samples. The area fraction of An# for each sample is plotted in Fig. 6. We divide plagioclase into two groups: (i) phenocryst plagioclase that was used for zone segmentation with crystal areas ≥ 32400 µm^2^ (81 pixels) and (ii) matrix plagioclase, with crystal areas < 32400 µm^2^ (81 pixels). Importantly, by using the method of Sheldrake and Higgins (2021), we ensure that mixed pixels (between plagioclase and another phase) do not contribute to the plagioclase chemical distributions in Fig. 6. Phenocrysts are typically euhedral and tabular, with broken crystals evidenced by discontinued zoning patterns in An#. Matrix plagioclase is present as acicular (e.g. SK385) as well as stubby, prismatic crystals (e.g. SK387).

The mean An# of all plagioclase crystals (phenocrysts + matrix) broadly increases vertically through the section (55–87), with units in the upper half displaying more pronounced bimodality in An# distributions (Fig. 6a). Whole rock chemistry (Fig. 4) and mean An# of all plagioclase are decoupled throughout such that samples with near-identical whole rock SiO_2_ (e.g. SK391 [61.8 wt%] and SK385 [61.4 wt%]; Fig. 6a) have contrasting An# distributions. Conversely, samples with different whole rock SiO_2_ (e.g. SK392 [50.6 wt%] and SK394A [60.3 wt%]; Fig. 6a) can exhibit similar mean and range of An#. Phenocryst plagioclase chemistry is also decoupled from whole rock chemistry (Fig. 4 and Fig. 6b), with the mean An# of phenocrysts consistently higher than the distributions of all plagioclase (Fig. 6a, b). Bimodality is still present in phenocryst plagioclase distributions and, in some cases, becomes more pronounced with respect to the distributions obtained considering all plagioclase (SK387, SK394A, SK394C; Fig. 6a, b). The sawtooth phenocryst distributions for SK385, SK386B and SK387 are a result of the smaller number of phenocrysts large enough for segmentation (Online Resource 7, Fig. S4). Rims of phenocrysts (defined as the outermost pixel of each crystal) are also translated towards higher An# and show wide, rather than unimodal, distributions that restrict in range towards the top of the section (Fig. 6c). Phenocryst rims do not converge towards a single value (or tighter range of values) compared with phenocryst plagioclase for a given eruption. Instead, wider phenocryst An# distributions produce proportionally wide phenocryst rim distributions, potentially reflecting exposure of disequilibrium cores and mantles in thin section due to crystal fracturing (see “Discussion”). In contrast to phenocrysts, matrix plagioclase shows unimodal distributions of An# with the exception of some wider tails up to An# 95 (Fig. 6d). Mean An# is consistently increasing with stratigraphic height, reaching a stable value of An# ~ 85 in the upper three units. SK408 An# distributions remain similar for both phenocryst and matrix plagioclase, retaining the tail to higher An#.

The complex, bimodal phenocryst distributions (Fig. 6b) reflect the composite phenocryst textures that vary widely between samples. The basal unit (SK408) contains euhedral phenocrysts, which are oscillatory zoned in the core and normally zoned in the rim, as well as normally zoned microphenocrysts (An# 50–90). SK385 has sparse phenocrysts (An# 70–90) that are predominantly weakly zoned with a sieve textured core. SK386B and SK387 show similar textural and chemical features in plagioclase. Both samples have an alignment of the acicular matrix plagioclase (An# 50–70), likely a result of bubble expansion during ascent (Degruyter et al., 2019). Phenocrysts are normally zoned, ranging from An# 70 to 95, with sieve texture in the mantle of some larger crystals. SK390 represents a transition to a larger proportion of high An# (90–98) plagioclase phenocrysts which exhibit normal zoning and a sieve textured mantle. SK391 has plagioclase with numerous melt inclusions and cracks, giving a “shredded” appearance in the phase maps (Online Resource 5, Fig. S2). Oscillatory zoning, occasionally initiating from an anorthitic core (85–90), is common. Unzoned high An# crystals are also present. SK392 contains abundant melt inclusions which are typically concentrated in the core or mantle but can be pervasive throughout the crystal. Phenocrysts have a wide variety of textures despite a relatively limited span of An# (75–95). Crystals can be homogeneous, displaying both normal and oscillatory zoning. Generally, the oscillatory zoned mantle–rim is smaller in areal extent than the homogeneous, high anorthite cores where they are present. SK394A has normally zoned, An# 80–95 plagioclase, with wide, high An# cores in the largest phenocrysts. However, in SK394C, the cores are, on average, lower in An# (85–90) compared with SK394A (excluding rare excursions to an An# ~ 95 mantle) and lower in the rims (An# 75 as opposed to An# 80).

**Fig. 7:**
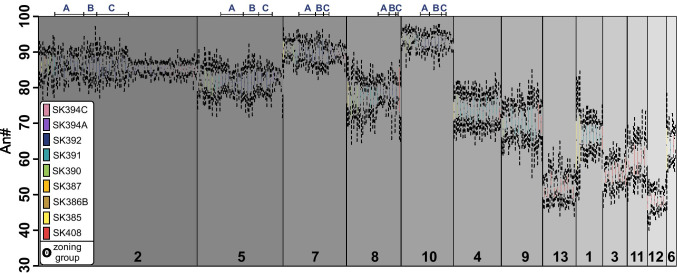
An# distributions of all segmented crystals, divided into 13 zoning groups. Zoning groups are ordered by decreasing abundance of crystals present in each group and coloured by sample. Dashed lines signify the interquartile range of each An# distribution. Three pieces of scoria were scanned for sample SK392 (A, B, C; upper *x*-axis labels)

Complex textures and zoning features that reappear frequently throughout the sequence suggest that plagioclase zoning may be correlated between samples. Figure 7 shows the results of the image segmentation technique whereby individual crystals are divided into chemical and textural zones. Each zone is then assigned to a zoning group on the basis of their similar An# distributions (e.g. Figure 3d). Samples vary with respect to the number of zoning groups present: some samples (e.g. SK408, SK391) are dominated by specific zoning groups whereas other samples (e.g. SK392, SK385) contain a wider array of zoning groups (Fig. 7). Zoning groups that include numerous samples (e.g. 2, 5, 7) are consistently higher in mean An#: likely these compositions reflect the dominant conditions of crystal growth within the magmatic system, which is ultimately revealed in the recurrence of samples containing the same zoning groups. Zoning group 10 is made up mostly of high An# cores found in rare phenocrysts of SK390, SK391, SK392, SK394A and SK394C (Fig. 7). Those samples with crystals which belong to multiple zoning groups (SK408, SK385, SK390, SK391) have appreciably wider An# distributions of phenocryst plagioclase (Fig. 6b).

**Fig. 8:**
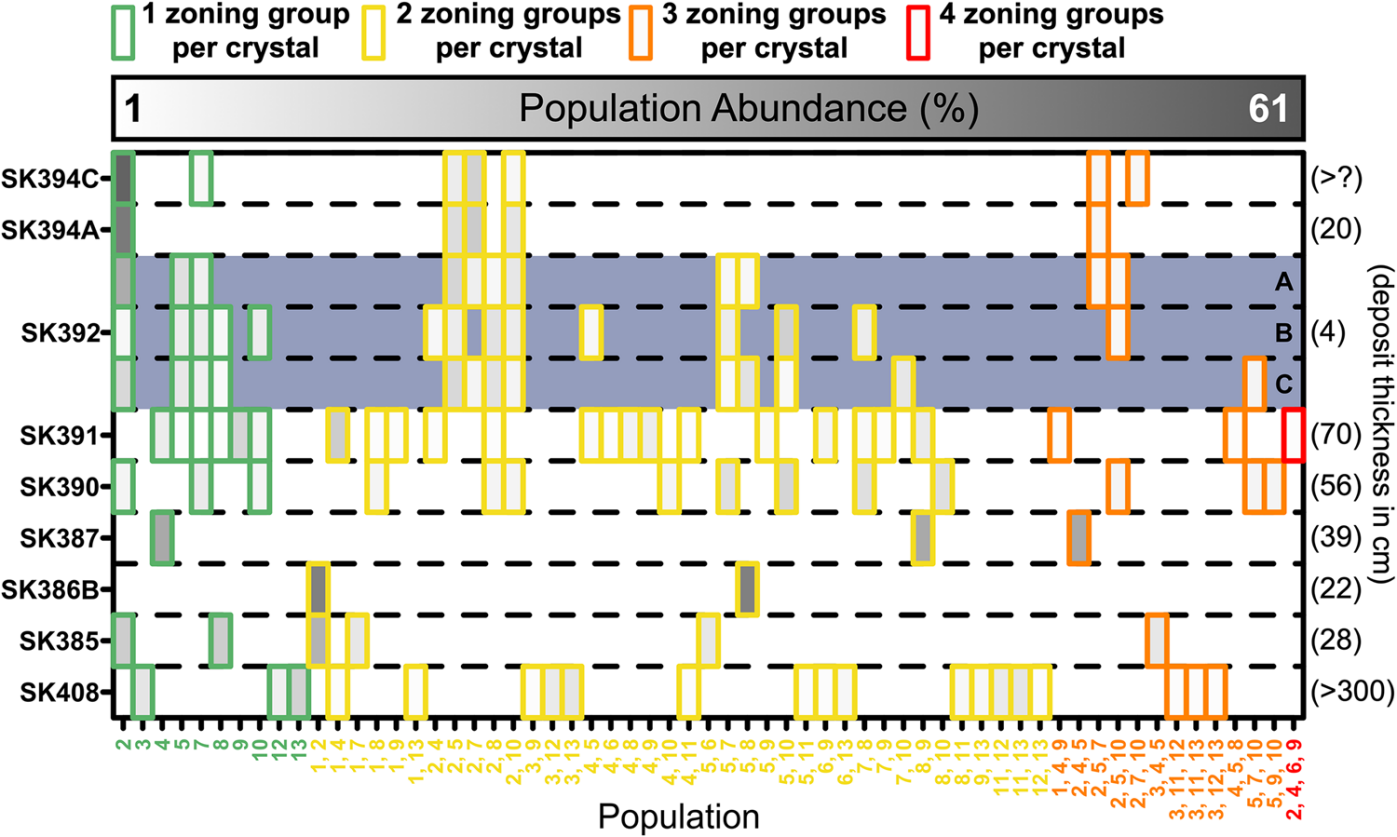
Population abundance for each sample plotted in stratigraphic order. A population is defined as crystals with the same combination of zoning groups (e.g. “1, 13” would be crystals composed of zoning group 1 + zoning group 13). Green, yellow, orange and red boxes denote 1, 2, 3 and 4 zoning groups per crystal, respectively. The interiors of boxes are shaded for abundance (% of crystals). SK394A and SK394C have amongst the lowest number of populations, with homogeneous crystals of zoning group 2 dominating in both samples. Right margin labels show volcanic deposit thickness (cm). In general, thicker deposits have a higher number of crystal populations. Note that the three samples of SK392 contain near-identical crystal populations even using relatively small scan areas (≤ 50 mm^2^) for the chemical maps

Crystal populations can be defined as groups of crystals that contain the same combination of zoning group(s) (Fig. 8). Each zoning group has been defined on the basis of both chemistry and texture. Thus, a single population represents crystals that have likely experienced the same combination of growth conditions within the magmatic system. There is an overall reduction in the number of populations per sample from the bottom to the top of the stratigraphy, with crystals comprised solely of zoning group 2 becoming more abundant with time. Each deposit contains a unique combination of populations, with some overlap in populations between samples. In general, the thicker deposits (e.g. SK391, SK390) comprise a higher number of populations than thinner deposits (e.g. SK385, SK394A; Fig. 8). The thickest deposits (including the basal pyroclastic flow deposit which was > 3 m thick with no base visible) contain a larger number of crystal populations composed of three and four zoning groups (orange and red boxes in Fig. 8).

## Discussion

### Whole rock–anorthite decoupling

Stratigraphic whole rock plots in certain systems (e.g. Figure 4) can highlight temporal geochemical patterns, which can be linked to volcanic and geochemical processes such as progressive or cyclical differentiation (Gertisser and Keller, 2003) or changes in eruptive style (Baker and Holland, 1973; Newhall, 1979; Roobol et al., 1985; Roobol and Smith, 1976). However, for the stratigraphic section we investigated, no regular pattern in the whole rock chemistry can be observed (Fig. 4). Whole rock trends instead show short wavelength cyclicity, largely uncorrelated with plagioclase An# (Figs. 4 and 6). The disconnect between whole rock and mineral chemistry has been noted in a variety of systems (including the intrusive environment; Latypov, 2015) and for a range of mineral phases (Charlier et al., 2005; Kahl et al., 2013; Stock et al., 2020; Ubide et al., 2014). Such a decoupling is unsurprising in the case of phenocrysts (Fig. 6b) considering that crystals in magmatic systems can experience contrasting, and often complex, histories before being incorporated into the same erupted magma volume prior to eruption (Davidson et al., 2007; Humphreys et al., 2006). Phenocrysts may have a hybrid origin, whereby part of the crystal is antecrystic (a crystal in equilibrium with an earlier pulse of magma which is incorporated into later pulses) and is then overgrown from the melt in which it is entrained. Unravelling the histories of these crystals requires extensive experimental work and/or thermodynamic modelling which can still recover non-unique P–T-X-H_2_O pathways (Cashman and Blundy, 2013).

However, matrix plagioclase crystals generally crystallise during the magma’s final assembly and ascent to the surface (Hammer and Rutherford, 2002) and are hence more likely to be in equilibrium with the melt. The anorthite content of plagioclase is largely modulated by changes in the temperature and water content of the melt, with hotter and wetter melts producing more An-rich plagioclase (Sisson and Grove, 1993). Moving up the stratigraphy, distributions of the matrix An# become tighter and mean values increase (Fig. 6d). This implies a progressive increase of temperature and thermal homogenisation within the magmatic plumbing system, noting the caveat that melt water content may also influence plagioclase chemistry. The large-volume basal unit (SK408; Pre-Mansion Pyroclastics) in the stratigraphy contains a wide range of phenocryst compositions, potentially reflecting the eruption of a large portion of an evolved magmatic reservoir. Hence, we suggest the lower units (A–C) of the Mansion Series, that overlay SK408, result from continued replenishment of the subvolcanic system of Saint Kitts. This has driven thermal and chemical homogenisation (Annen et al., 2006) and a progressive increase in the proportion of mafic mineral compositions in the subvolcanic reservoir (Weber et al., 2020; Fig. 5 and Fig. 6). Such a clear and continuous increase in An# of plagioclase through time is rarely noted in volcanic environments. However, it is a common feature of layered intrusions and mafic sills, presenting in so-called mafic reversals. Similar to our explanation above, such reversals may occur when magmas become more primitive with time (e.g. the Vavukansky dolerite sill; Latypov and Egorova, 2012).

Considering the matrix plagioclase An# as a proxy for the matrix chemistry, the fact that there is no clear relationship between whole rock and matrix plagioclase highlights the extent to which whole rock chemistry tends to mask continuous or cyclical processes. In general, this issue can also extend to matrix glass (e.g. Pantelleria, Italy), where measured glass chemistry may span a significantly wider chemical spectrum than the whole rock chemistry (Liszewska et al., 2018; Williams et al., 2014). The extreme of this is so-called “cryptic melts” where, even in chemical systems that are remarkably homogeneous in their bulk rock characteristics, diverse mineral chemistry unveils hidden melt compositions (Stock et al., 2020). Based on the decupling observable on Saint Kitts, we would advocate for caution when interpreting magmatic processes based on bulk rock chemistry. In fact, we highlight the elegant study of Ubide et al., (2014) who demonstrate the extent to which entrained crystal cargo can heavily distort interpretations of the primitive nature of lamprophyres. In andesitic samples in particular (e.g. Saint Kitts), where whole rock and melt inclusion chemistry are generally compositionally distinct (Reubi and Blundy, 2009), we subscribe to the view that phenocrysts may be the exception rather than the rule (Ubide et al., 2014).

The high An# tails in the chemical distribution of matrix plagioclase (Fig. 6d) may either represent entrained crystals, mixed populations of matrix plagioclase, or be the consequence of fragmentation of the more anorthitic phenocrysts. The latter is consistent with both the fragmented nature of pyroclastic rocks in general (Miwa and Geshi, 2012; Taddeucci et al., 2021) and the wide distributions of phenocryst rims (Fig. 6c), potentially reflecting exposure of mantles and cores in disequilibrium with the matrix glass. To explore this further, we quantify the degree of fragmentation of the phenocrysts for each sample. This first requires a validation of the image segmentation method followed by an analysis of the phenocryst zoning patterns.

### Intra-unit correlation

The robustness of the segmentation technique was explored using sample SK392, a mafic (50.6 wt% SiO_2_) scoria layer. In order to map a total area of > 100 mm^2^, three separate scoria pieces from the deposit were individually scanned, effectively representing three random samples of the same eruption. Segmentation identified the same five zoning groups in all three scoriae independently (Fig. 7), despite their small scan areas (50 mm^2^, 25 mm^2^ and 25 mm^2^, respectively), as well as several commonalities in crystal populations (Fig. 8). All scoriae are similar in both their populations present and the abundances of each population. SK394A, a subsequent eruption, has plagioclase composed of most of the same zoning groups as SK392. However, SK394A has fewer populations (5; Fig. 8) than SK392, despite some overlap in certain populations between the two samples (e.g. population 2,5 and population 2,10). SK394A is dominated by homogeneous population 2 crystals, and many crystal populations composed of multiple zoning groups contain zoning group 2 (Fig. 8). In contrast, SK392 has notably more populations composed of two zoning groups per crystal compared to SK394A, although homogeneous population 2 crystals are also present in SK392.

The reproducibility of both zoning groups and populations for a single eruption (SK392A–C), as well as the contrasting populations observed in a subsequent event (SK394A), suggests that the crystal populations present, and their proportions, provide a unique fingerprint for samples of a given volcanic eruption (Fig. 8). Hence, the segmentation approach we present here may also prove useful for the correlation of tephra layers between outcrops. We would expect the similarity between samples of the same eruption to converge as the scan area increases, i.e. more crystals quantified. Furthermore, duplication of crystal textures between scoriae from the same sample (SK392) implies a reproducible process that is collecting a unique combination of zoned crystals from within the magmatic plumbing system prior to eruption. This supports the current understanding of a lensed magmatic system composed of pockets of melt and crystals whereby one eruption can be derived from a given melt lens (Christopher et al., 2015). Some crystals may be obtained from the melt lens itself and some may be entrained during ascent to the surface, but this process is duplicatable and likely path dependent for a given event. These lenses may each be variably differentiated and mixed before final shallow storage and eruption (Cooper et al., 2019).

### Variability in the complexity of plagioclase phenocrysts between eruptive units

Following the verification of the method for a single sample, we can now examine how crystal populations change through time between eruptions. Figure 8 demonstrates that populations are shared between multiple volcanic units, and that certain populations may disappear and reappear throughout the stratigraphy. We found no volcanic unit which is represented by a single population, despite the segmentation algorithm theoretically allowing this, supporting the idea that phenocrysts have experienced multiple thermo-chemical conditions of growth prior to eruption.

Considering the number of populations present in a given sample (Fig. 8) as a proxy for textural complexity reveals an evolution throughout the stratigraphic sequence that is not evident from whole rock data alone. In conjunction with An# distributions (Fig. 6), this serves to identify dominant crystal assemblages. The progressive increase in matrix An# (Fig. 6d) is coupled with a broad decrease in the overall textural complexity of plagioclase phenocrysts in time, with 20 populations present in the basal unit and only 7 at the top of the sequence (Fig. 8). Additionally, there is a transition from crystals distributed between multiple populations (SK408) to homogeneous population 2 crystals becoming by far the most dominant (SK394A and SK394C; Fig. 8). In fact, population 2 crystals are the most common for the sequence as a whole (Fig. 8), present in 5 of the 9 stratigraphic units. We suggest that population 2 crystals reflect the dominant magma in the volcanic system based on their ubiquity throughout the sequence as well as their high abundance and homogeneity in the upper units, which contain the most anorthitic plagioclase. These upper units are mineralogically “clean” in that they contain tight chemical distributions of matrix plagioclase, and phenocryst rims that match closely to matrix chemistry (Fig. 6c, d).

Superimposed on this overall reduction in complexity is a dependence between the bed thickness and the textural complexity, such that thicker beds (SK390, SK391, SK408) contain the highest number of crystal populations. Additionally, the thickest units (SK390, SK391, SK408) contain the highest number of populations composed of three and four zoning groups per crystal (e.g. population 5,9,10 and population 2,4,6,9; Fig. 8). Hence, there appears to be a combination of two competing effects influencing the textural complexity of crystals in the erupted products. One is the chemical and physical heterogeneity within the pre-eruptive storage region sampled by an eruption. The other is the erupted volume, assuming that bed thickness is proportional to eruption magnitude, with larger eruptions sampling greater regions of the magmatic plumbing system and hence a greater variety of crystals (phenocrysts or antecrysts). Whilst there may be some variability of deposit thickness between different outcrops of the same section around the Saint Kitts coastline due to dispersal characteristics, based on deposit mapping (Roobol et al., 1985), this effect is minor at such close proximity to the source and so the qualitative relationship between complexity and thickness should hold. The occurrence of larger events midway through the stratigraphy (SK390, SK391) implies that eruption magnitude is not controlled by the progressive thermal evolution observed in An# (Discussion, above), at least at millennial timescales.

### Integrating physical volcanology and petrology using mineral zoning patterns

**Fig. 9:**
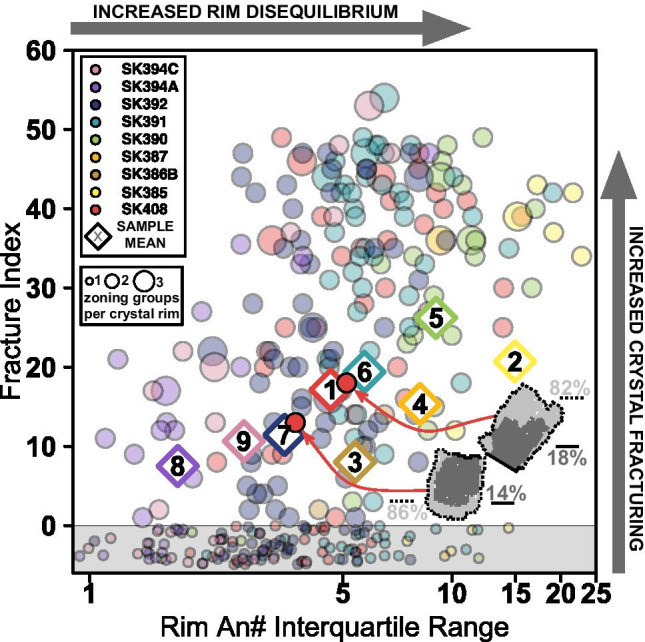
Fracture index (FI) vs rim An# interquartile range of all phenocrysts (crystals subjected to zone segmentation). Crystals growing with concentric zoning patterns should have their youngest zone surrounding the crystal margin, irrespective of cut section effects (Fig. 3 of Cheng et al., 2017). One mechanism for this condition not being satisfied is that the crystal is fractured, exposing core or mantle zones on the outer margin of the crystal. This process is evident in thin section and chemical maps from Saint Kitts samples, as well as in the exemplar crystal from SK408 (inset of this figure). The fracture index is defined as 100 minus the percentage of the most abundant zoning group that occupies the rim of each crystal (Eq. 1). The rim is defined as the outermost pixel of each crystal. The greater the fracture index, the higher the likelihood of crystal fracturing. Those crystals with FI = 0 are all single zoning group crystals (crystals with FI = 0 have been jittered in the Y direction for clarity and lie within the grey-shaded area). There is a correlation between the mean FI (diamonds) and the rim An# interquartile range, suggesting scatter in An# of phenocryst rims (Fig. 6c) is largely being controlled by crystal fracturing. Numbers inside diamonds relate to relative stratigraphic order of the samples (1 = oldest, 9 = youngest)

A key aim of igneous petrology is to use chemical and textural information from rocks and minerals to infer magmatic processes occurring at depth. In addition to our approach of segmenting mineral zoning patterns, another parameter we can extract from our data is a quantification of crystal fracturing. A consequence of stereology is that, irrespective of the cut orientation of a crystal, the outermost zone should occupy the whole exterior of a crystal (Fig. 3 of Cheng et al., 2017). The thickness of this rim is unlikely to be uniform due to cut orientation as well as preferential growth on certain crystallographic faces (Holness, 2014). Many phenocrysts from Saint Kitts with two or more zoning groups per crystal show incomplete growth of a single exterior zoning group and, in many cases, the cutting of zoning patterns is evident in An# maps as well as thin section (e.g. Figure 3). A prime example can be seen in the inset of Fig. 9 where a broken crystal from SK408 would show a continuous exterior zoning group if it was not split in half. Hence, the rim of every crystal should, in theory, be represented by a single zoning group as per the segmentation method. Deviation from this geometry can be related to crystal fracturing or disequilibrium textures such as incomplete late-stage resorption of outer zones. As such, we have defined the fracture index (FI) as

1 $$\mathrm{F}\mathrm{I}=100-{X}_{r}$$

where *X*_r_ is the percentage of the outer rim that is assigned to the most abundant zoning group (i.e. the higher the FI, the more fractured the crystal, where the rim is the outermost pixel of the phenocryst from the chemical maps).

In all samples FI and the rim An# interquartile range are positively correlated, suggesting that fracturing exposes the inner portions of the phenocrysts, effectively increasing the compositional range of the outer rims (Fig. 6c and Fig. 9). There is also a relationship between the presence of tails in matrix An# distributions (Fig. 6d) and the phenocryst sample mean values (Fig. 9; large diamonds). SK390 and SK387 (higher FI, more fractured) have wide matrix tails whereas SK394A, SK394C and SK386B (lower FI, less fractured) have narrower distributions. This suggests fragments of phenocrysts are producing these tails and that the modal value of An# rather than the range of its distribution is more representative of the matrix plagioclase chemistry. Potential mechanisms of crystal fracturing include a brittle response to stress perturbations in the magmatic system, melt inclusion decrepitation, bubble expansion or shearing in the conduit (Taddeucci et al., 2021; van Zalinge et al., 2018).

## Conclusions

The quantified chemical mapping of plagioclase has allowed us to interrogate magmatic processes with a greater statistical significance compared to whole rock chemistry and EPMA spot analyses alone. Further subdividing crystals on the basis of their size and textural relationships (phenocryst, matrix, rim; Fig. 6b–d) reveals a clear disparity between phenocryst plagioclase and matrix plagioclase. Matrix plagioclase presents a progressive transition to less-evolved compositions in time (higher An#; Fig. 6d). Understanding the timescales over which this increase, and eventual saturation at high An# (An# ~ 85), is repeated on Saint Kitts could be used as a predictive metric for the length of eruption cycles. This pattern is largely independent of whole rock trends which reveal more chaotic fluctuations (Fig. 4).

The 2D textural segmentation of phenocrysts has identified 13 different zoning groups throughout the stratigraphy and 61 crystal populations (Figs. 7 and 8). Zoning segmentation shows a decrease in textural complexity of plagioclase towards the top of the section as well as an increase of homogeneous and high-anorthite population 2 crystals (Figs. 6 and 8). Together, these quantitative parameters suggest the contribution of less-evolved compositions to the erupted magma increases through the sequence, progressing towards a more homogeneous subvolcanic reservoir (Figs. 6 and 8). Additionally, textural complexity correlates with deposit thickness, such that the thickest deposits have the highest complexity. This intimates that higher volume eruptions have a propensity to sample larger, more heterogeneous regions of the magmatic plumbing system.

In the future, quantifying the proportion of antecrystic versus phenocrystic crystal populations (Neave et al., 2017) may be possible by combining the results of this study with a similar analysis of plagioclase from the concomitant plutonic nodules erupted on Saint Kitts (Macdonald et al., 2000; Melekhova et al., 2017). In general, applying the segmentation method to plutonic systems may be useful to understand the dynamics in the roots of active volcanoes as phenocryst zoning patterns tend to be better correlated in plutonic, compared to volcanic, environments (Pietranik et al., 2006). Further information may be gained by combining the results of major element segmentation with zoning patterns from trace element mapping (Ubide et al., 2015).

The ubiquity of plagioclase in volcanic rocks means the segmentation method could become a powerful and widely applicable tool for tephrostratigraphy and mapping of volcanic units that are difficult to correlate via conventional methods. However, this would require robust testing to understand the statistical reproducibility of populations using variations in scan area and crystal number. Furthermore, applying the segmentation approach to other volcanoes could provide new insights into relationships between textural complexity, fracture index and eruptive dynamics. Understanding how these parameters vary within a stratigraphic context could impart essential information to appreciate temporal dynamics of magmatic systems and better anticipate the eruptive behaviour of volcanoes.

## Supplementary Information

Below is the link to the electronic supplementary material.

Supplementary file1 (DOCX 18 KB)

Supplementary file2 (PDF 25401 KB)

Supplementary file3 (DOCX 23 KB)

Supplementary file4 (XLSX 48 KB)

Supplementary file5 (PDF 1453 KB)

Supplementary file6 (PDF 821 KB)

Supplementary file7 (PDF 2478 KB)

Supplementary file8 (PDF 3950 KB)

## Data Availability

All data required will be made available.
