# Supplementary material for: Quantitative chemical mapping of plagioclase as a tool for the interpretation of volcanic stratigraphy: an example from Saint Kitts, Lesser Antilles
Source: Bull Volcanol. 2021 Jul 16;83(8):51. doi: 10.1007/s00445-021-01476-x (PMC8549933; doi:10.1007/s00445-021-01476-x)
Supplement: Supplementary file 1 — Supplementary file1 (DOCX 18 KB) [file 445_2021_1476_MOESM1_ESM.docx]

| **Sample** | **Thickness (cm)** | **General Description** |
| --- | --- | --- |
|  |  |  |
|  |  |  |
| **SK394C** | NA | Volcanic bomb from top of SK394 layer |
| SK394B | 15 |  |
| **SK394A** | 20 | Angular to sub angular grey pumice layer. Rich in lithics. Potential fining upwards sequence |
| SK393B | 4 | Green, microvesicular pumice |
| SK393A | 4 | Fine, dark grey lapilli |
| NA | 54 | Series of lapilli, ash and pumice. Interbedded black mafic scoria with brown soily ash |
| **SK392** | 4 | Black angular – sub angular pumices |
| NA | 30 | Fine, brown layer of ash with small pumices |
| **SK391** | 70 | Pumice rich layer. White pumices. |
| **SK390** | 56 | Tan coloured, ash-rich deposit containing pumices and dark grey lithics. |
| NA | 4 | Surge layer of grey ash |
| SK389C | 11 | Grey-brown ash rich layer. Lapilli < 2cm |
| SK389B | 3 | Grey-brown lapilli and ash deposit |
| NA | 15 | Brown, ash-rich layer. Phreatic?? |
| NA | 9 | Grey lapilli and ash deposit, sub rounded pumice < 2cm |
| SK389A | 66 | Ash rich lapilli pumice, Grey-brown colour, sub rounded pumices |
| NA | 25 | Grey volcanic deposit. Lapilli, angular clasts < 2 cm in size. |
| SK388 | 43 | Angular brown clasts with microporosity. Juvenile pumices with small cumulate inclusions |
| **SK387** | 39 | Slight coarsening upwards sequence. Juveniles |
| **SK386B** | 22 | Coarsening upwards sequence. Juveniles have microporosity, light grey-green colour. Sparsely porphyritic. Juveniles are mm – 3-4 cm in size. |
| SK386A | 12 | Very fine ash with small pumices. Grey colour. Small pieces of lapilli sized material. Some yellow, hydrothermally altered material. |
|  |  | Mafic layer with reworked beach sand. Angular, light grey, microvesicular pumice. Coarsening upwards from a few mm at the base to centimeter size at the top. Juveniles have large crystals of amphibole. |
| **SK385** | 28 |  |
| **SK408** | NA | Poorly sorted white pumice with grey lithics. Pumice are cm – mm scale. Lithics have a large range in size up to the metre scale. Clots of mafic minerals |

**Table S1** Unit descriptions for all samples within the stratigraphic log. Samples with designated sample numbers have been analysed for major and trace element in the bulk rock (**Online Resource 3 - Table S2**). Samples in bold have been selected for chemical mapping and plagioclase segmentation. Thickness denotes the thickness of each stratigraphic layer

**Quantitative chemical mapping of plagioclase as a tool for the interpretation of volcanic stratigraphy: an example from Saint Kitts, Lesser Antilles**

*Bulletin of Volcanology* **(Online Resource 1)**

**Oliver Higgins*, Tom Sheldrake, Luca Caricchi**

Department of Earth Sciences, University of Geneva, rue des Maraîchers 13, 1205, Geneva, Switzerland

*Corresponding author (oliver.higgins@unige.ch; ORCID iD: 0000-0001-9960-934X)
