# Supplementary material for: Quantitative chemical mapping of plagioclase as a tool for the interpretation of volcanic stratigraphy: an example from Saint Kitts, Lesser Antilles
Source: Bull Volcanol. 2021 Jul 16;83(8):51. doi: 10.1007/s00445-021-01476-x (PMC8549933; doi:10.1007/s00445-021-01476-x)
Supplement: Supplementary file 2 — Supplementary file2 (PDF 25401 KB) [file 445_2021_1476_MOESM2_ESM.pdf]

Quantitative chemical mapping of plagioclase as a tool for the interpretation of volcanic stratigraphy: an example from Saint Kitts, Lesser Antilles

Bulletin of Volcanology **(Online Resource 2)**

Oliver Higgins*, Tom Sheldrake, Luca Caricchi
Department of Earth Sciences, University of Geneva, rue des Maraîchers 13, 1205, Geneva, Switzerland
*Corresponding author (oliver.higgins@unige.ch; ORCID iD: 0000-0001-9960-934X)

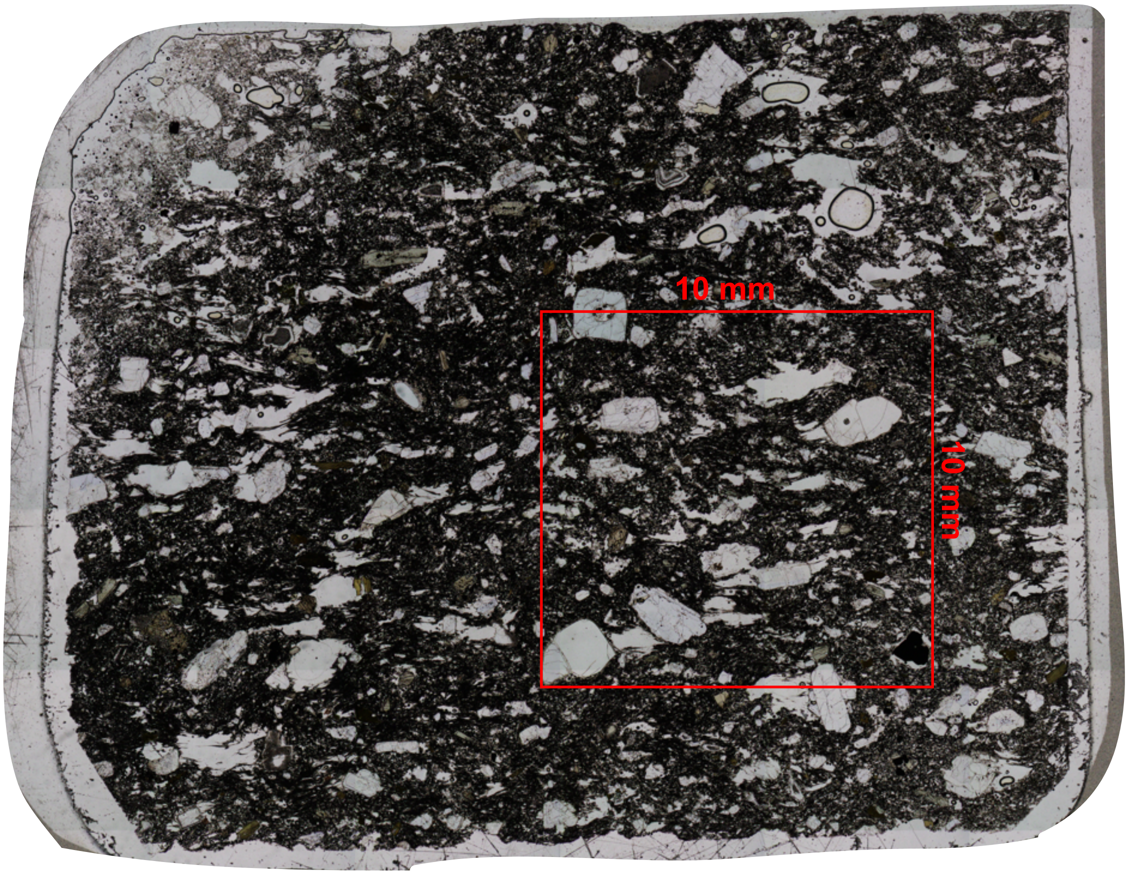

SK408

SK385

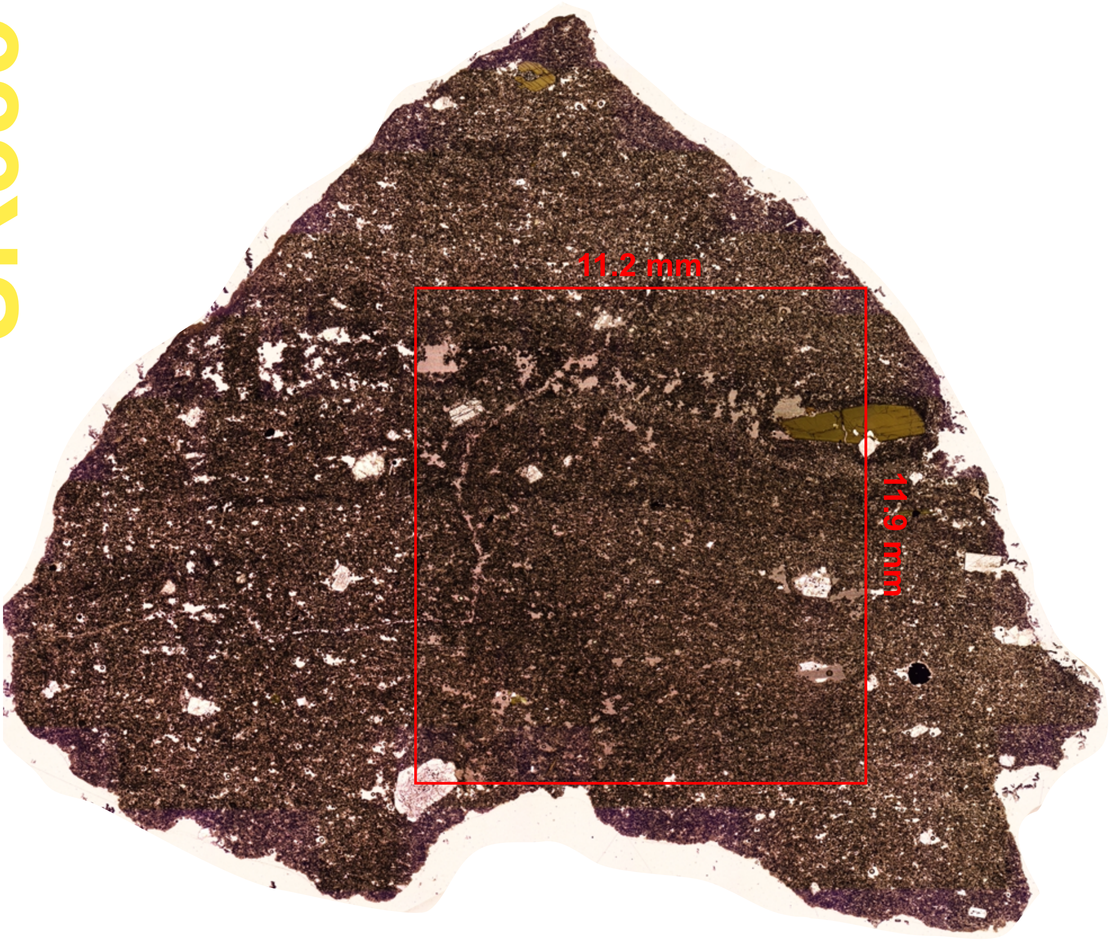

SK386B

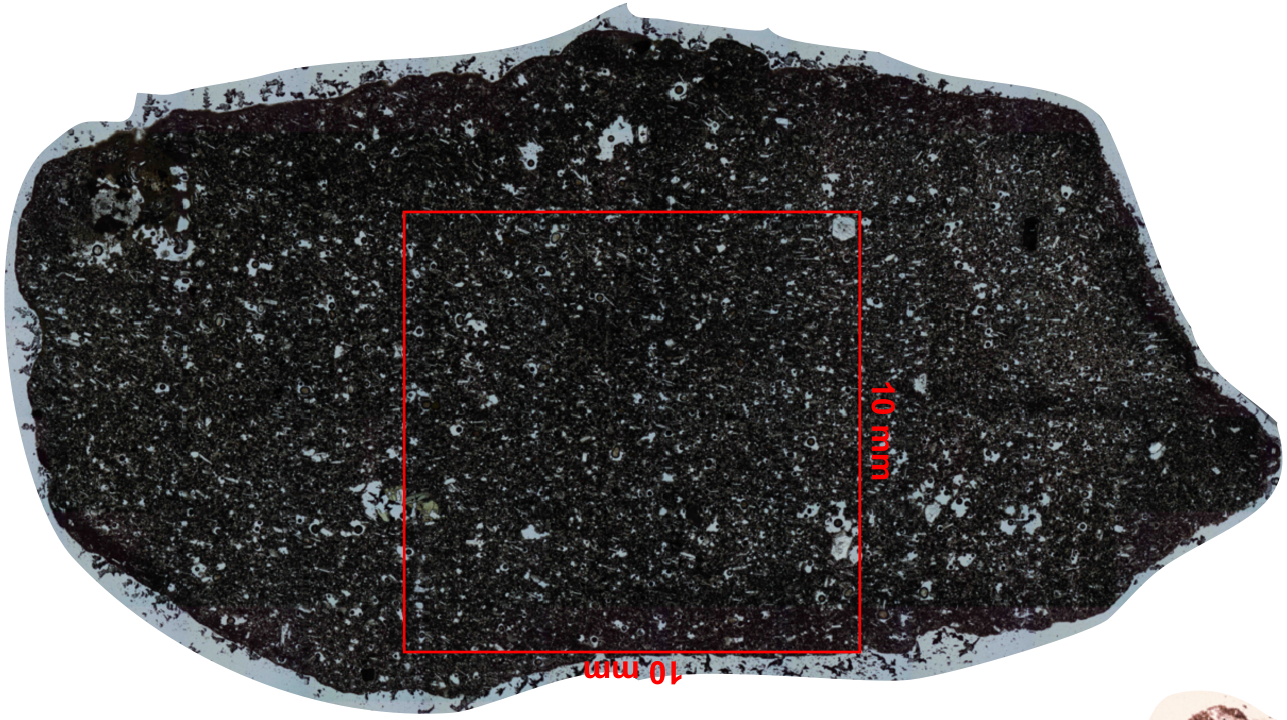

SK387

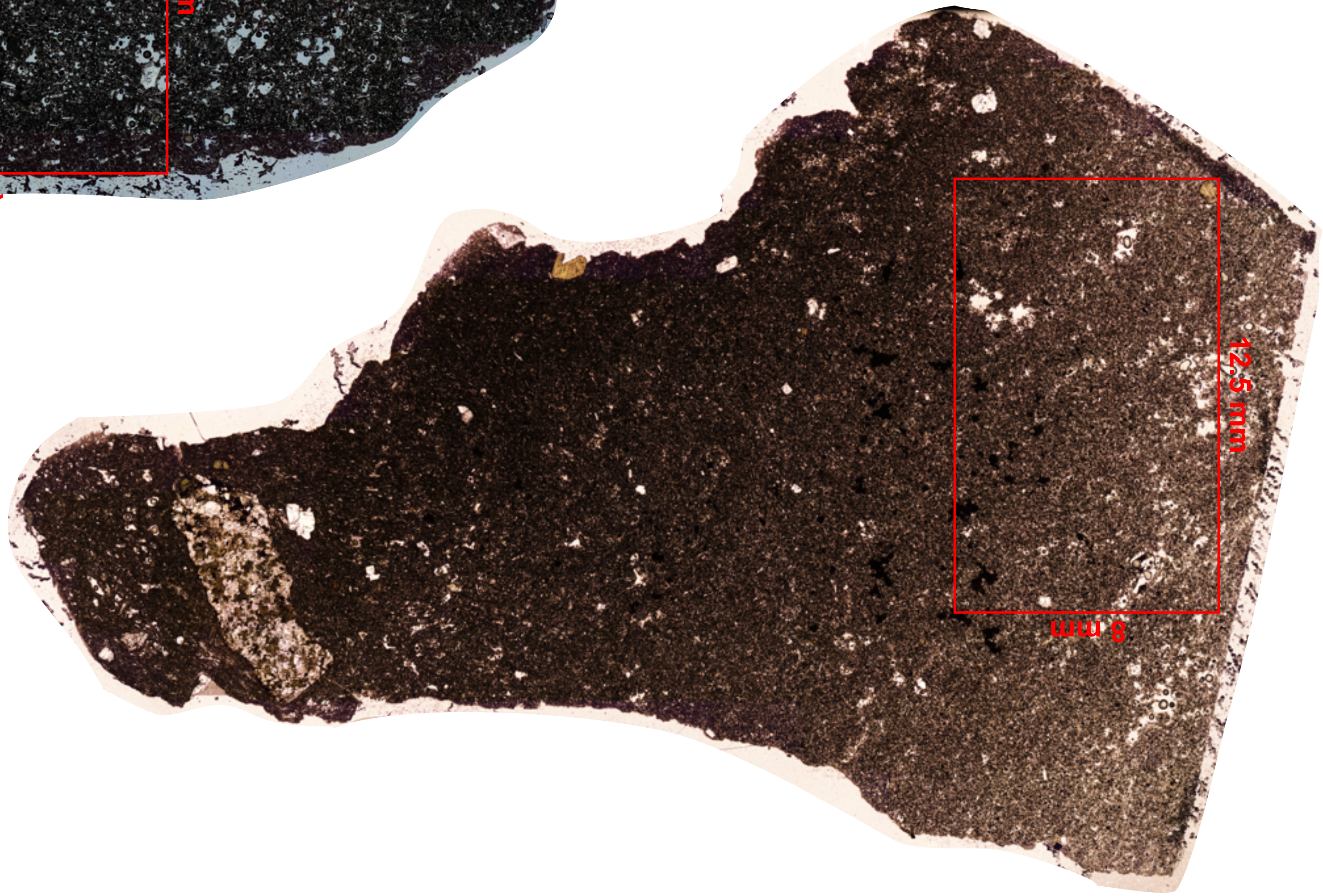

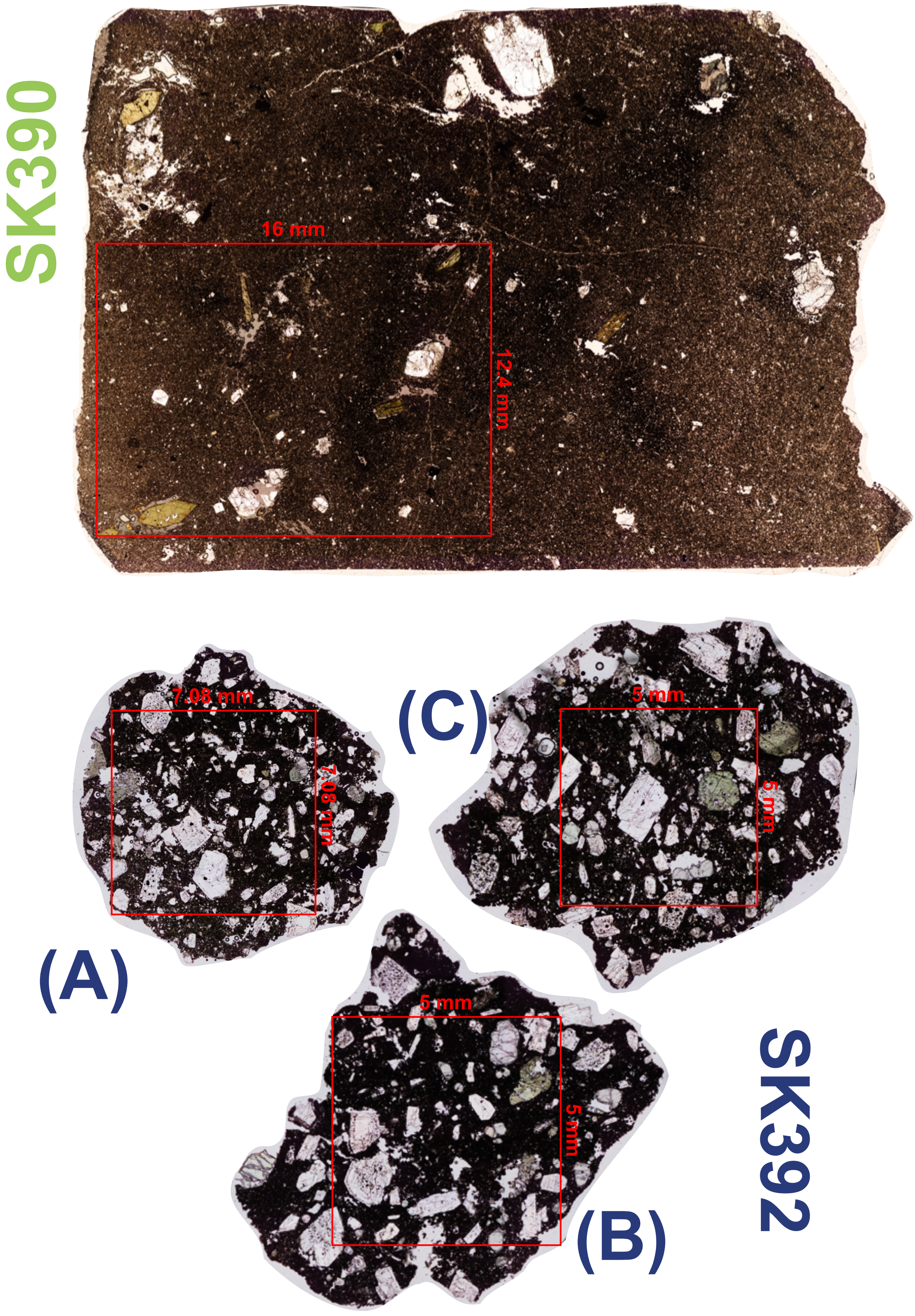
SK390
16 mm
12.4 mm
7.08 mm
7.08 mm
(A)
(C)
5 mm
5 mm
5 mm
5 mm
(B)
SK392

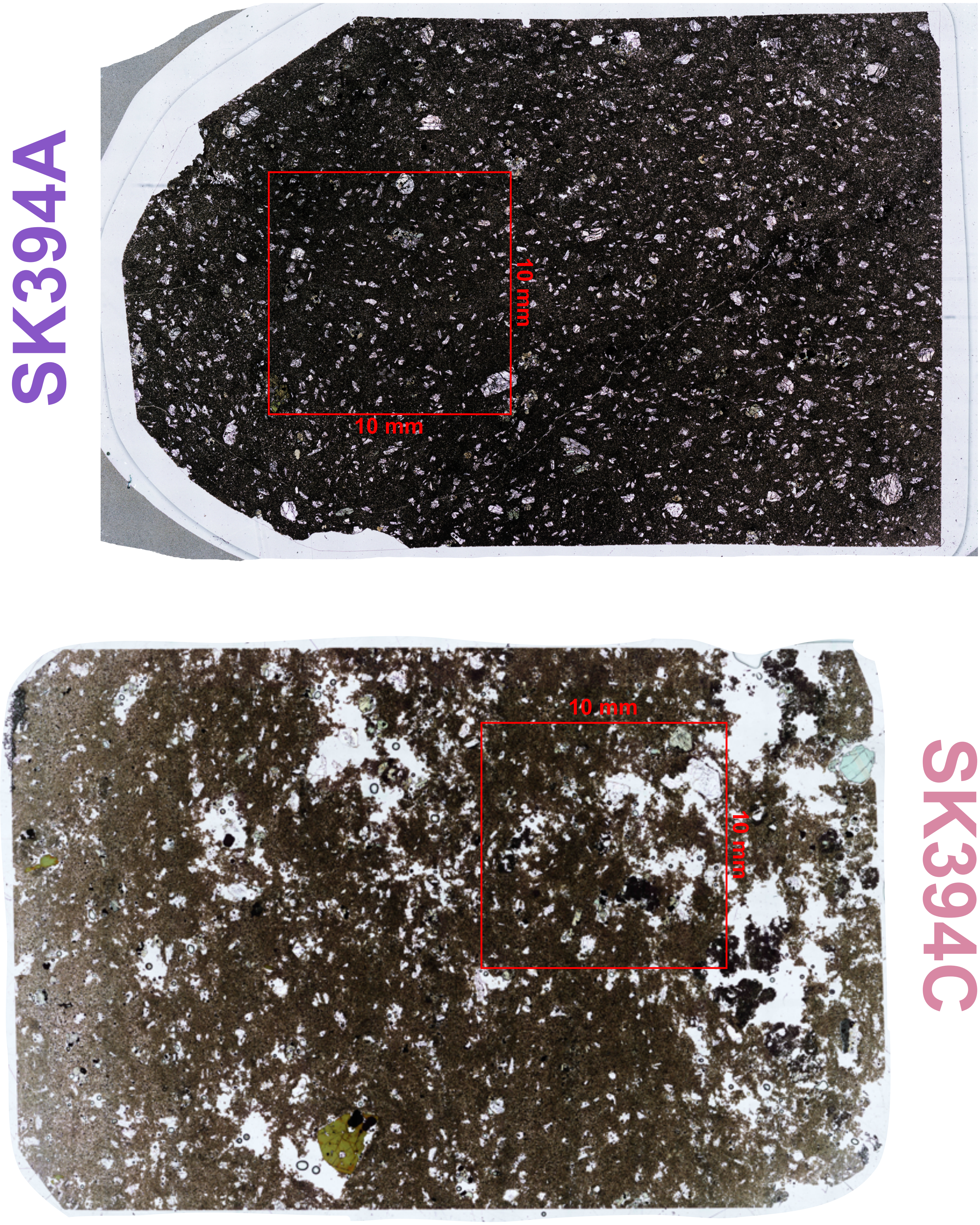

Fig. S1 Thin section scans in plane polarised light of samples used in this study (excluding SK391 which is on a circular mount). Red rectangles show areas selected for chemical mapping
