# Supplementary material for: Quantitative chemical mapping of plagioclase as a tool for the interpretation of volcanic stratigraphy: an example from Saint Kitts, Lesser Antilles
Source: Bull Volcanol. 2021 Jul 16;83(8):51. doi: 10.1007/s00445-021-01476-x (PMC8549933; doi:10.1007/s00445-021-01476-x)
Supplement: Supplementary file 3 — Supplementary file3 (DOCX 23 KB) [file 445_2021_1476_MOESM3_ESM.docx]

**Quantitative chemical mapping of plagioclase as a tool for the interpretation of volcanic stratigraphy: an example from Saint Kitts, Lesser Antilles**

*Bulletin of Volcanology* **(Online Resource 3)**

**Oliver Higgins*, Tom Sheldrake, Luca Caricchi**

Department of Earth Sciences, University of Geneva, rue des Maraîchers 13, 1205, Geneva, Switzerland

*Corresponding author (oliver.higgins@unige.ch; ORCID iD: 0000-0001-9960-934X)

| **Sample** | SK394C | SK393A | SK392 | SK391 | SK390 | SK389C | SK389B | SK389A | SK388 | SK386B | SK386A | SK385 | SK408 |
| --- | --- | --- | --- | --- | --- | --- | --- | --- | --- | --- | --- | --- | --- |
|  |  |  |  |  |  |  |  |  |  |  |  |  |  |
| **SiO_2_ (wt%)** | 60.75 | 54.38 | 50.63 | 61.77 | 57.96 | 55.94 | 56.43 | 55.81 | 58.98 | 60.50 | 56.61 | 61.36 | 63.30 |
| **TiO_2_ (wt%)** | 0.53 | 0.74 | 0.80 | 0.49 | 0.60 | 0.69 | 0.62 | 0.64 | 0.56 | 0.52 | 0.54 | 0.46 | 0.49 |
| **Al_2_O_3_ (wt%)** | 17.90 | 20.11 | 18.89 | 17.05 | 17.73 | 18.39 | 17.21 | 17.79 | 17.94 | 17.45 | 16.64 | 17.47 | 16.29 |
| **FeO (wt%)** | 7.08 | 8.20 | 9.21 | 6.09 | 8.41 | 8.32 | 7.93 | 8.04 | 8.14 | 7.59 | 7.13 | 7.47 | 6.04 |
| **MnO (wt%)** | 0.21 | 0.17 | 0.17 | 0.18 | 0.24 | 0.20 | 0.21 | 0.20 | 0.23 | 0.22 | 0.20 | 0.23 | 0.16 |
| **MgO (wt%)** | 2.00 | 3.50 | 6.44 | 1.89 | 2.89 | 3.68 | 3.10 | 3.43 | 2.53 | 2.12 | 2.72 | 1.88 | 2.35 |
| **CaO (wt%)** | 6.68 | 9.11 | 10.98 | 6.30 | 7.47 | 8.28 | 9.76 | 9.88 | 7.20 | 6.46 | 11.64 | 6.27 | 5.69 |
| **Na_2_O (wt%)** | 4.01 | 3.16 | 2.39 | 5.11 | 3.95 | 3.73 | 3.91 | 3.49 | 3.67 | 4.32 | 3.68 | 4.08 | 4.47 |
| **K_2_O (wt%)** | 0.69 | 0.51 | 0.40 | 0.98 | 0.62 | 0.65 | 0.68 | 0.58 | 0.60 | 0.64 | 0.67 | 0.58 | 1.09 |
| **P_2_O_5_ (wt%)** | 0.16 | 0.11 | 0.08 | 0.15 | 0.13 | 0.11 | 0.14 | 0.13 | 0.15 | 0.18 | 0.17 | 0.19 | 0.11 |
| **Sc (ppm)** | 11.6 | 21.7 | 40.9 | 12.9 | 13.7 | 18.9 | 14.9 | 17.3 | 8.6 | 10.6 | 13.4 | 9.8 | 13.9 |
| **Ni (ppm)** | 2.4 | 6.1 | 14.5 | 2.4 | 4.0 | 5.5 | 3.6 | 5.3 | 2.8 | 2.4 | 2.8 | 3.0 | 2.7 |
| **Rb (ppm)** | 13.6 | 8.8 | 7.0 | 16.6 | 10.3 | 10.7 | 11.1 | 10.1 | 11.7 | 10.6 | 11.1 | 12.2 | 18.4 |
| **Sr (ppm)** | 264.9 | 266.8 | 215.0 | 227.2 | 253.1 | 260.8 | 278.5 | 289.7 | 270.6 | 270.3 | 377.6 | 271.0 | 226.0 |
| **Y (ppm)** | 22.2 | 19.4 | 16.9 | 27.8 | 22.8 | 21.0 | 21.5 | 20.3 | 20.9 | 21.1 | 20.8 | 21.1 | 21.3 |
| **Zr (ppm)** | 75.7 | 56.4 | 44.7 | 103.6 | 72.5 | 65.6 | 72.6 | 65.2 | 69.7 | 75.4 | 75.3 | 75.4 | 97.2 |
| **Nb (ppm)** | 1.4 | 1.1 | 0.8 | 1.7 | 1.3 | 1.3 | 1.4 | 1.4 | 1.4 | 1.8 | 1.7 | 1.6 | 1.8 |
| **Ba (ppm)** | 134.9 | 100.3 | 72.5 | 168.2 | 108.9 | 120.1 | 133.4 | 119.9 | 113.8 | 122.1 | 141.2 | 110.5 | 206.4 |
| **La (ppm)** | 4.6 | 3.4 | 2.6 | 6.7 | 4.3 | 4.4 | 4.5 | 4.2 | 4.3 | 5.0 | 4.9 | 4.7 | 6.7 |
| **Nd (ppm)** | 9.2 | 7.2 | 5.4 | 11.6 | 8.9 | 8.4 | 8.6 | 8.3 | 8.9 | 9.6 | 9.1 | 9.4 | 10.5 |
| **Eu (ppm)** | 1.0 | 0.8 | 0.7 | 1.0 | 1.0 | 0.9 | 0.9 | 0.9 | 0.9 | 1.0 | 0.9 | 1.0 | 0.9 |
| **Dy (ppm)** | 3.6 | 3.3 | 3.0 | 4.5 | 3.8 | 3.5 | 3.5 | 3.4 | 3.4 | 3.6 | 3.5 | 3.5 | 3.4 |
| **Yb (ppm)** | 2.7 | 2.3 | 1.9 | 3.3 | 2.8 | 2.4 | 2.6 | 2.4 | 2.4 | 2.6 | 2.5 | 2.6 | 2.6 |
| **Lu (ppm)** | 0.4 | 0.3 | 0.3 | 0.5 | 0.4 | 0.4 | 0.4 | 0.4 | 0.4 | 0.4 | 0.4 | 0.4 | 0.4 |
| **Hf (ppm)** | 2.2 | 1.7 | 1.3 | 3.1 | 2.2 | 2.0 | 2.1 | 2.0 | 2.0 | 2.1 | 2.2 | 2.1 | 2.9 |

**Table S2** Selected bulk rock major (wt%) and trace elements (ppm) for all volcanic deposits in the studied stratigraphic section
