# Supplementary material for: Quantitative chemical mapping of plagioclase as a tool for the interpretation of volcanic stratigraphy: an example from Saint Kitts, Lesser Antilles
Source: Bull Volcanol. 2021 Jul 16;83(8):51. doi: 10.1007/s00445-021-01476-x (PMC8549933; doi:10.1007/s00445-021-01476-x)

Quantitative chemical mapping of plagioclase as a tool for the interpretation of volcanic stratigraphy: an example from Saint Kitts, Lesser Antilles

Bulletin of Volcanology **(Online Resource 5)**

Oliver Higgins*, Tom Sheldrake, Luca Caricchi
Department of Earth Sciences, University of Geneva, rue des Maraîchers 13, 1205, Geneva, Switzerland
*Corresponding author (oliver.higgins@unige.ch; ORCID iD: 0000-0001-9960-934X)

Fig. S2 Phase maps of chemically mapped samples derived using the phase separation approach of Sheldrake and Higgins (2021)

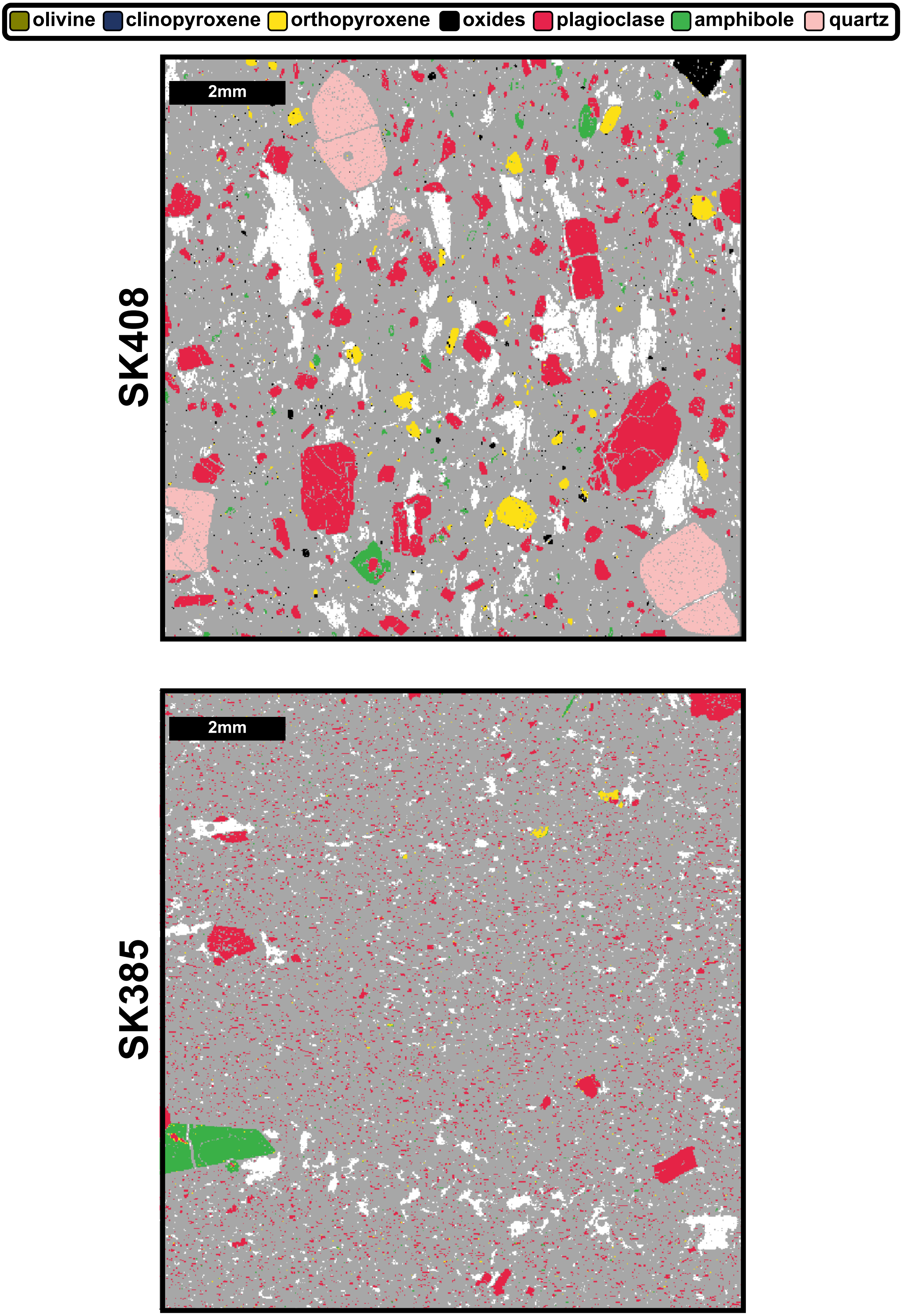
olivine
clinopyroxene
orthopyroxene
oxides
plagioclase
amphibole
quartz
2mm
SK408
2mm
SK385

SK386B

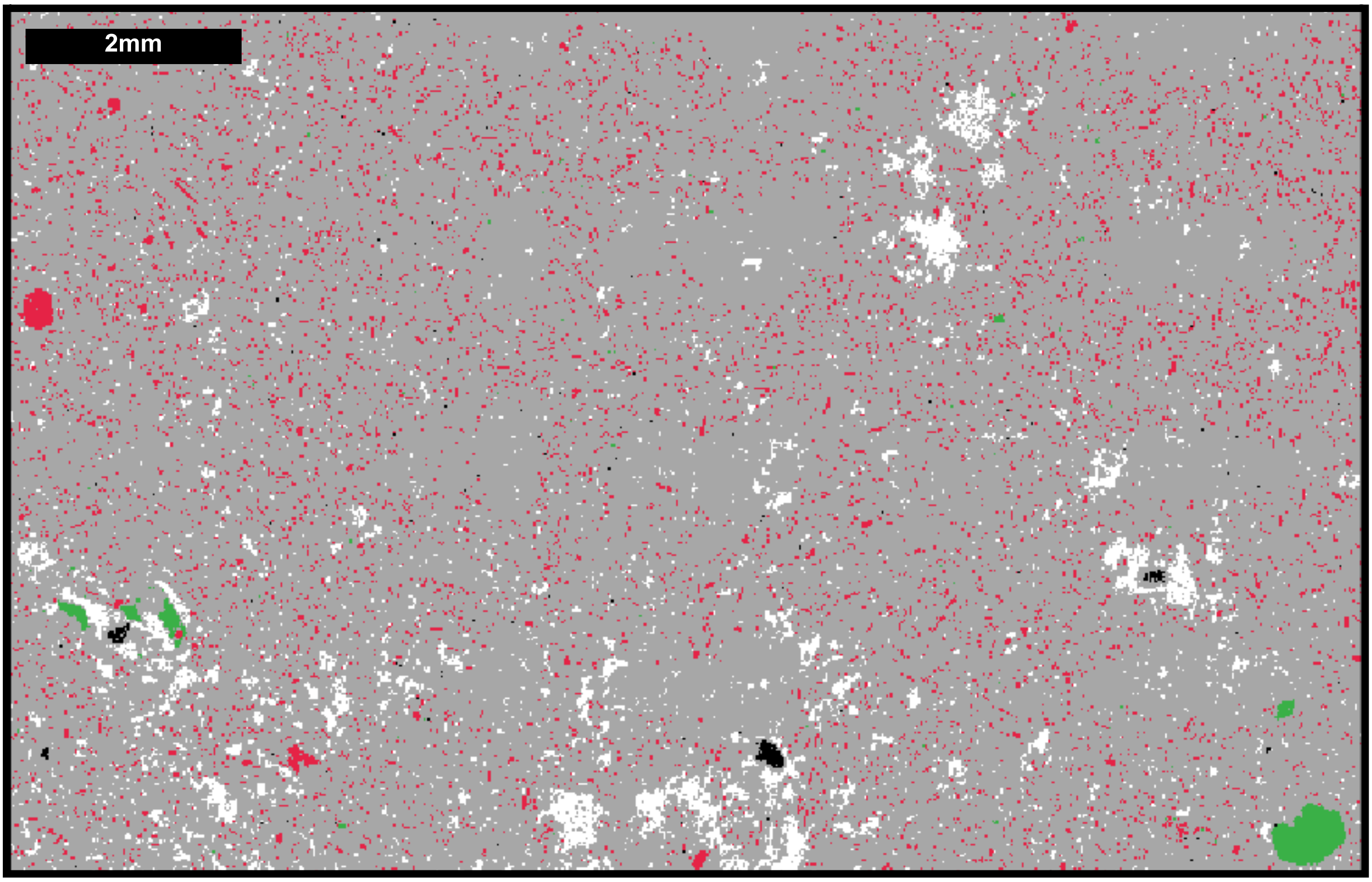

SK387

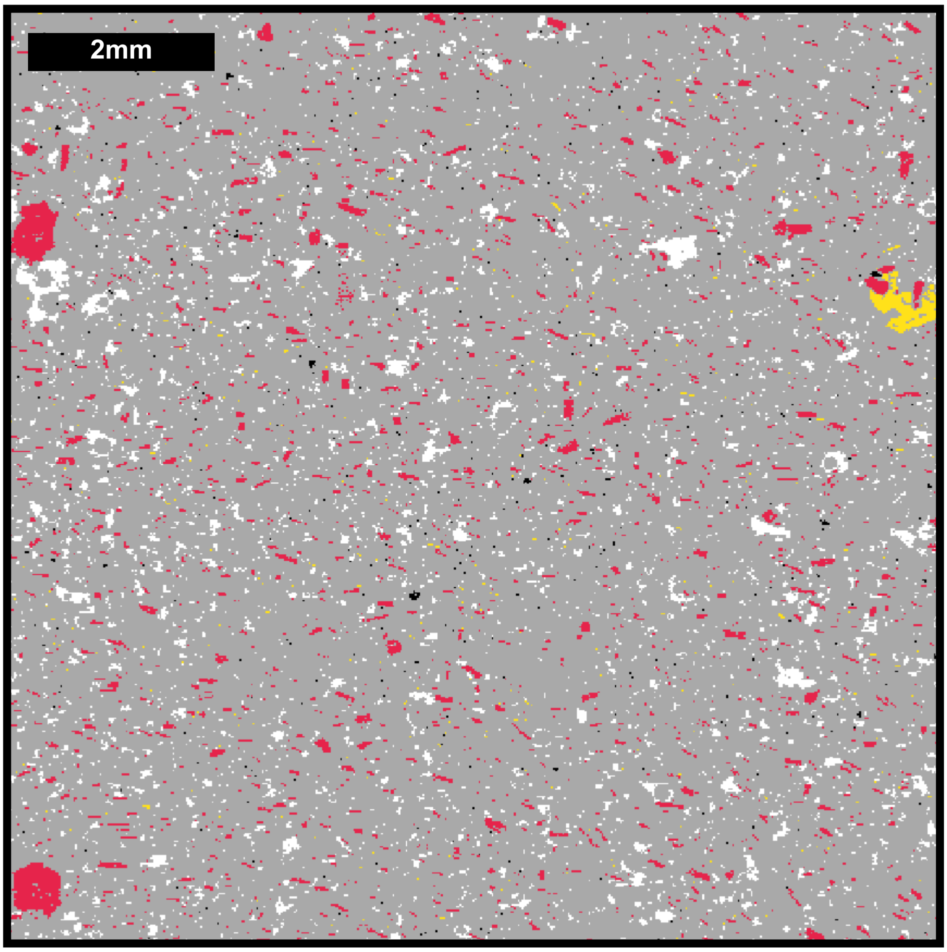

SK390

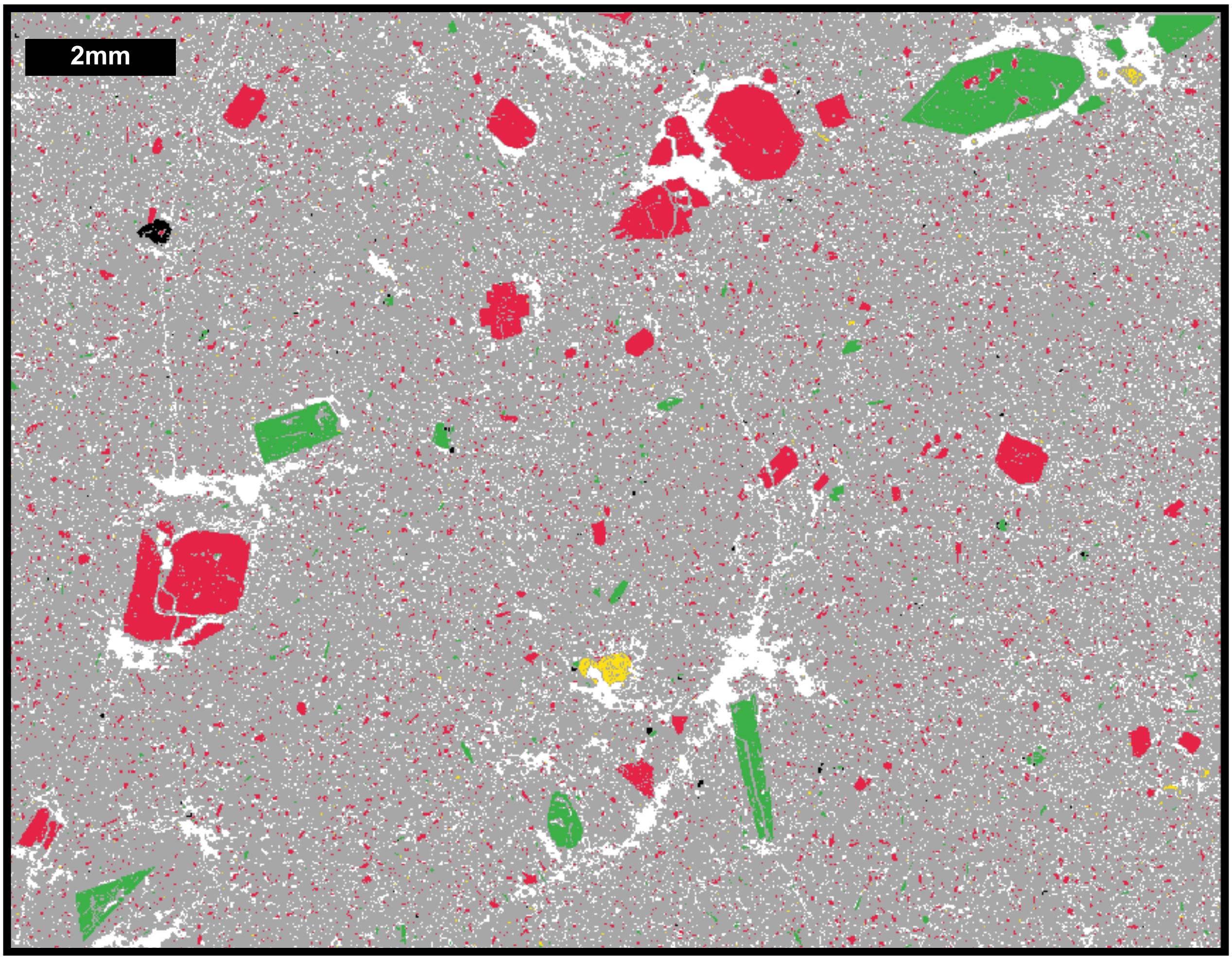

SK391

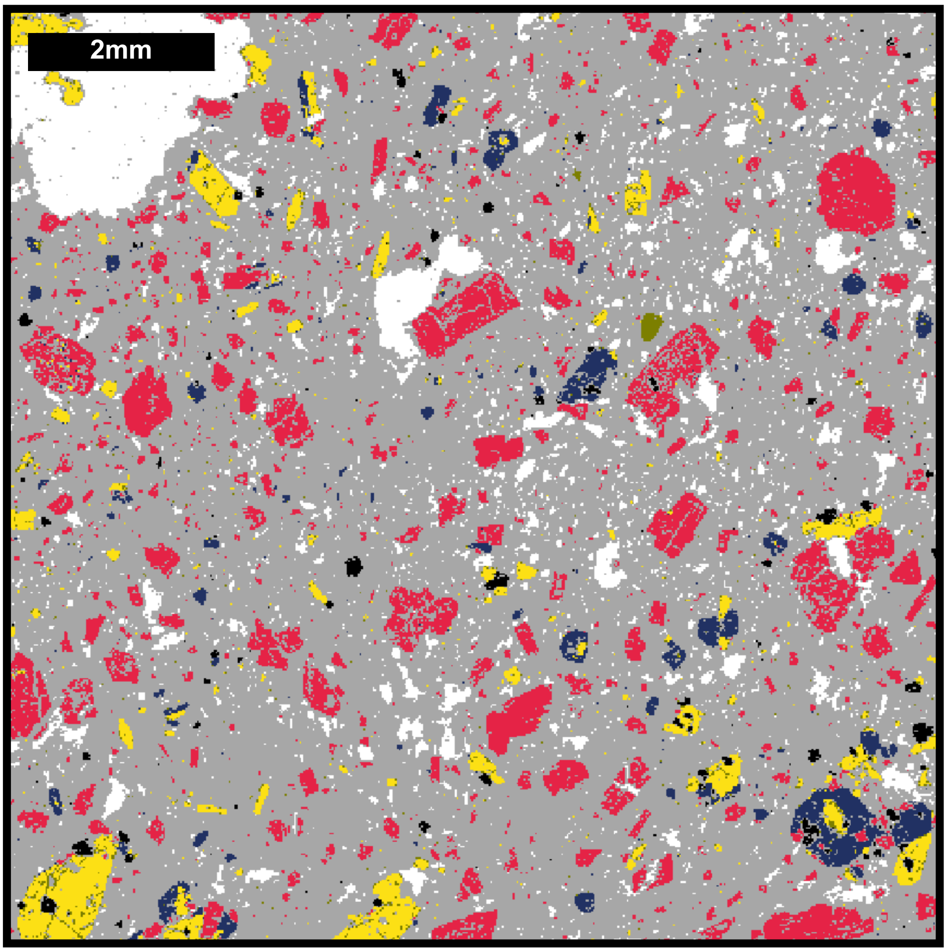

SK392 (A)

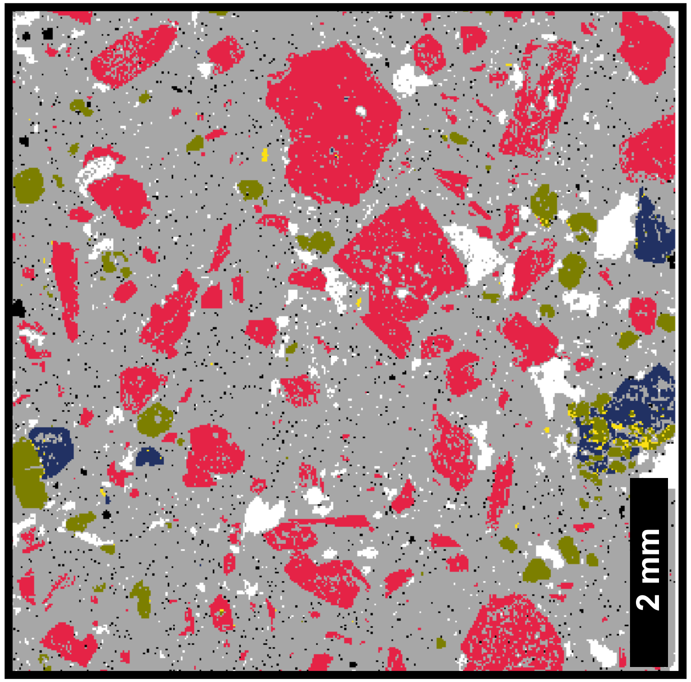

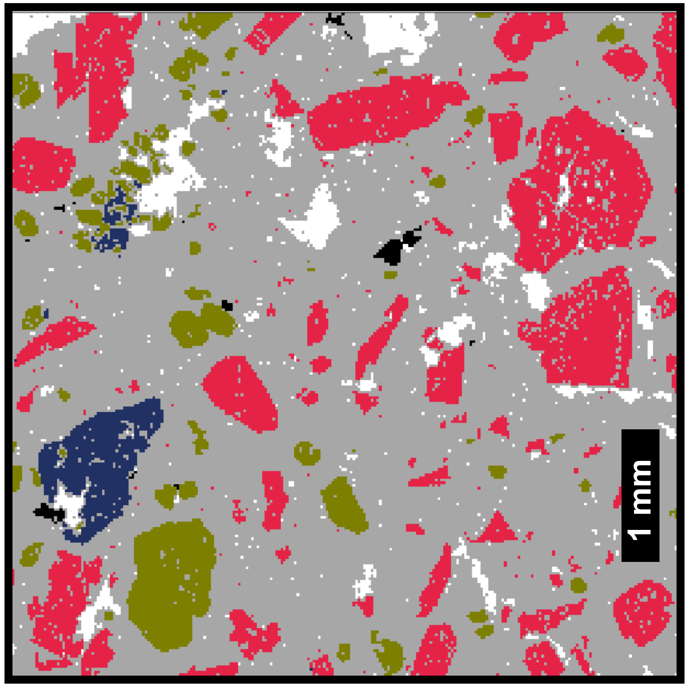

SK392 (B)

SK392 (C)

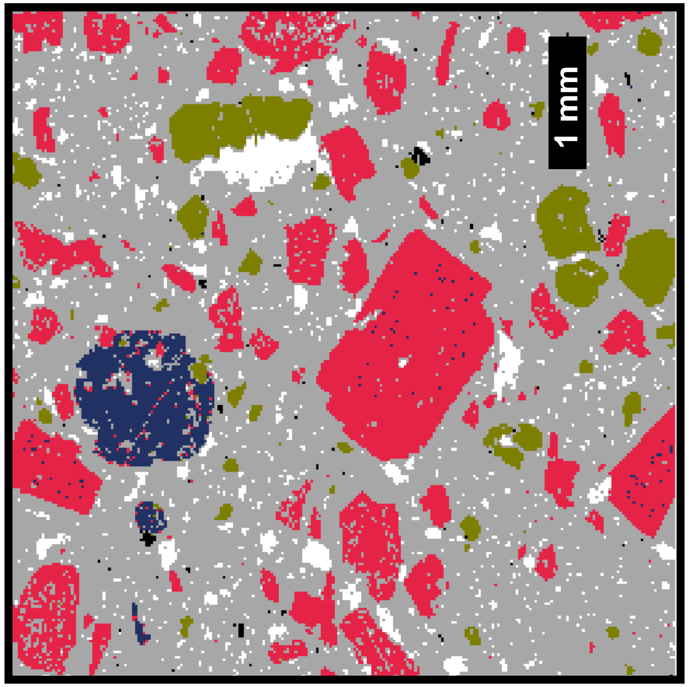

SK394A

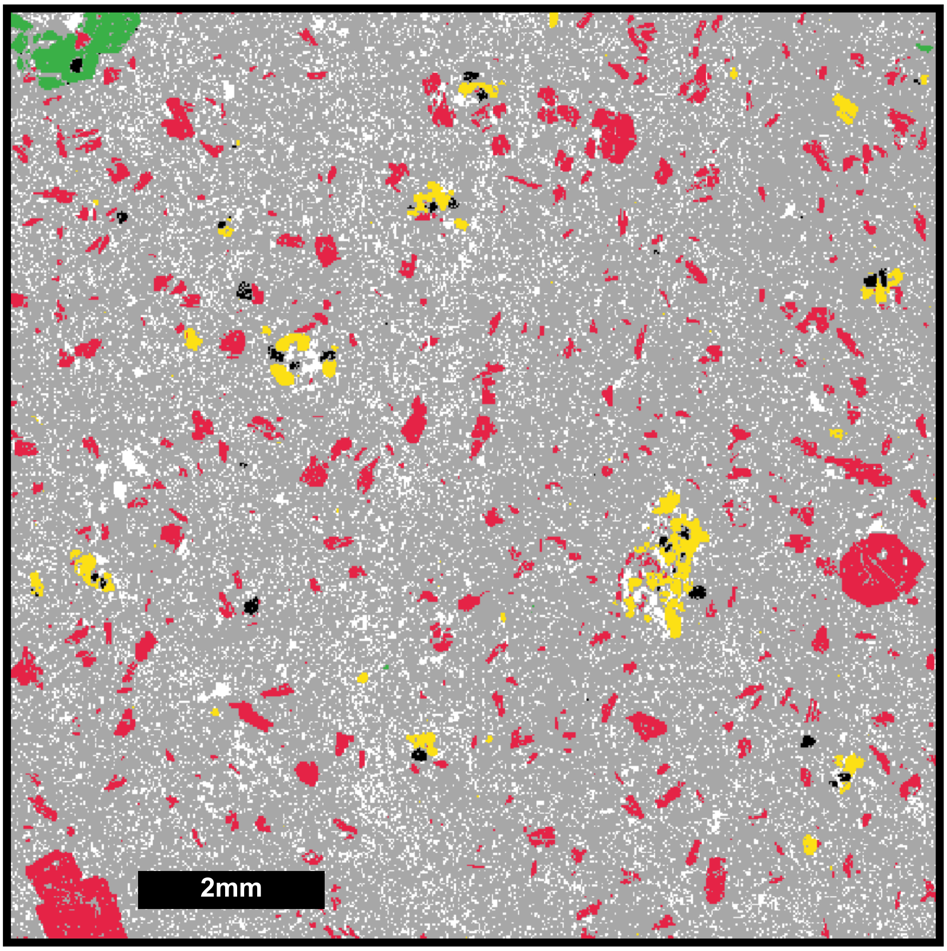

SK394C

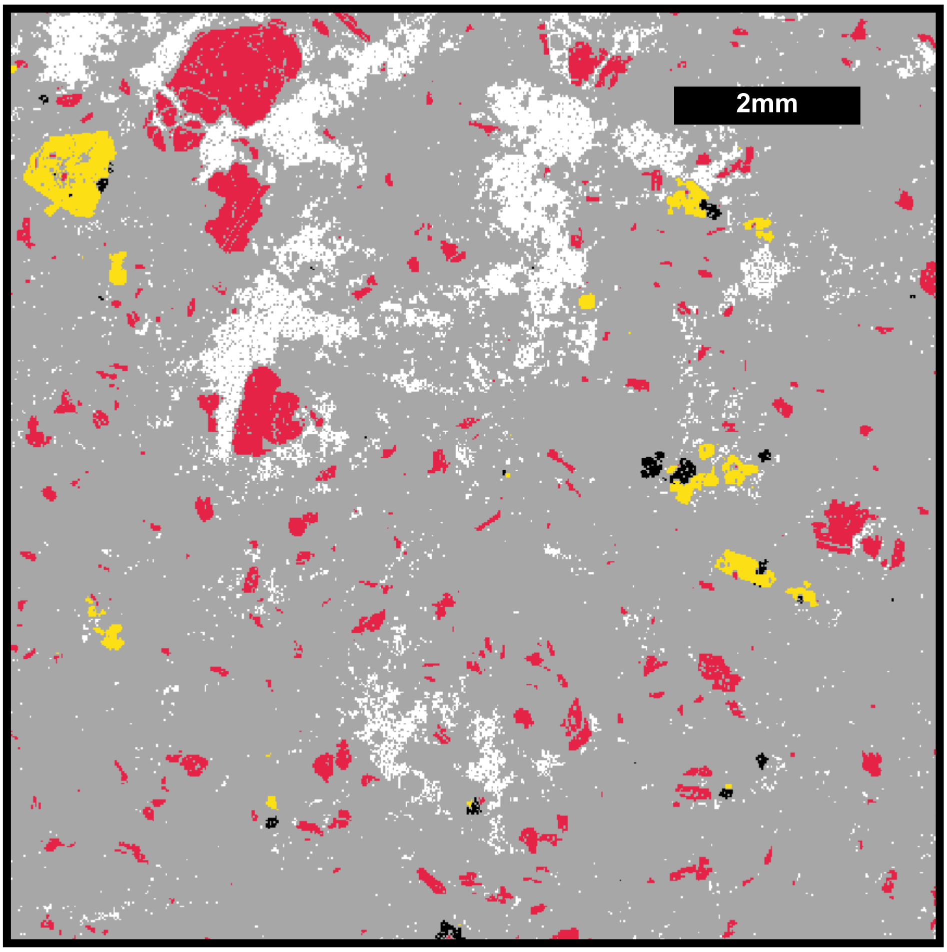

Supplement: Supplementary file 5 — Supplementary file5 (PDF 1453 KB) [file 445_2021_1476_MOESM5_ESM.pdf]
