# Supplementary material for: Quantitative chemical mapping of plagioclase as a tool for the interpretation of volcanic stratigraphy: an example from Saint Kitts, Lesser Antilles
Source: Bull Volcanol. 2021 Jul 16;83(8):51. doi: 10.1007/s00445-021-01476-x (PMC8549933; doi:10.1007/s00445-021-01476-x)
Supplement: Supplementary file 6 — Supplementary file6 (PDF 821 KB) [file 445_2021_1476_MOESM6_ESM.pdf]

Quantitative chemical mapping of plagioclase as a tool for the interpretation of volcanic stratigraphy: an example from Saint Kitts, Lesser Antilles

Bulletin of Volcanology **(Online Resource 6)**

Oliver Higgins*, Tom Sheldrake, Luca Caricchi
Department of Earth Sciences, University of Geneva, rue des Maraîchers 13, 1205, Geneva, Switzerland
*Corresponding author (oliver.higgins@unige.ch; ORCID iD: 0000-0001-9960-934X)

Fig. S3 Calibrated An# maps of all plagioclase (phenocryst + matrix) from the samples used in this study for segmentation

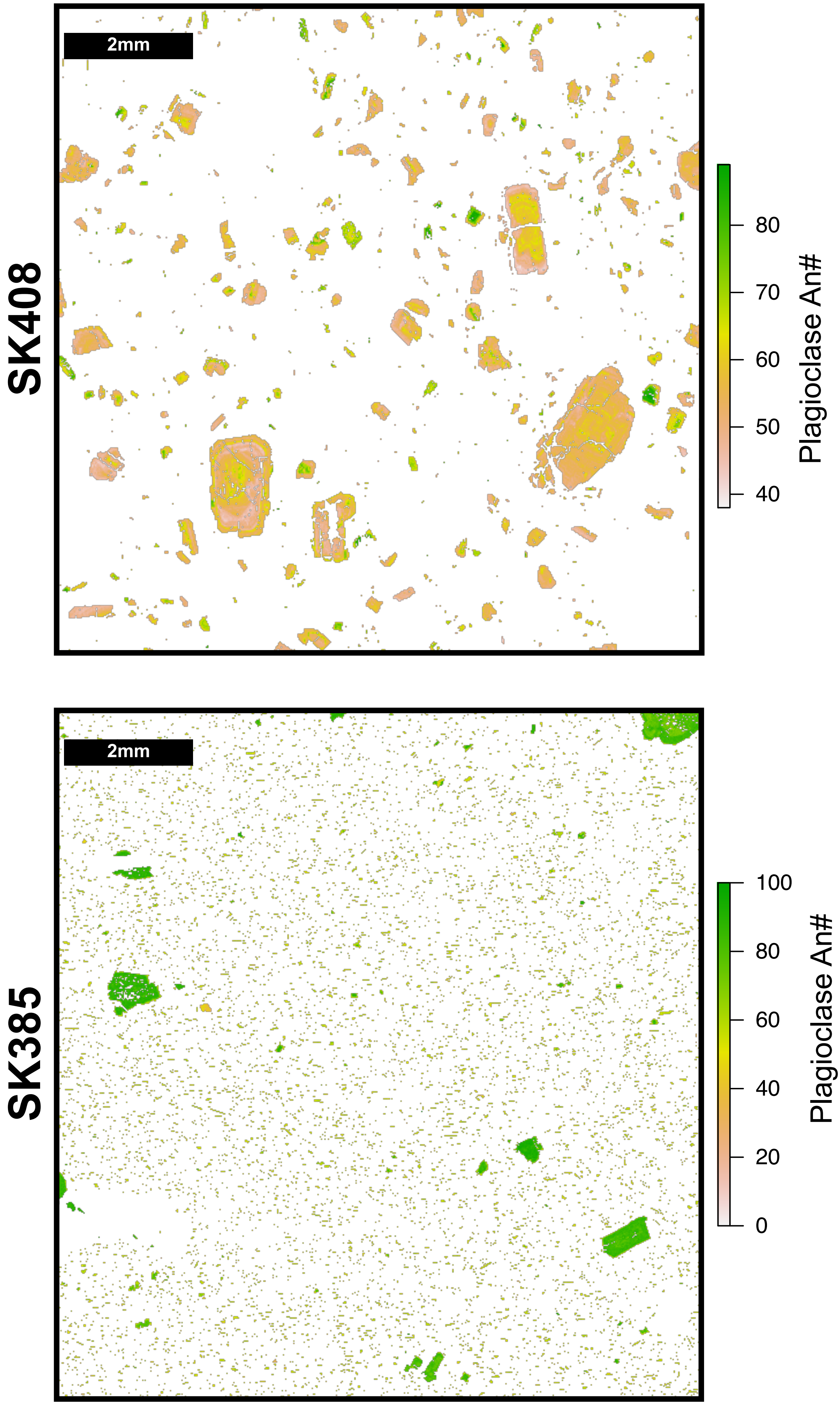
SK408
2mm
80
70
60
50
40
Plagioclase An#
SK385
2mm
100
80
60
40
20
0
Plagioclase An#

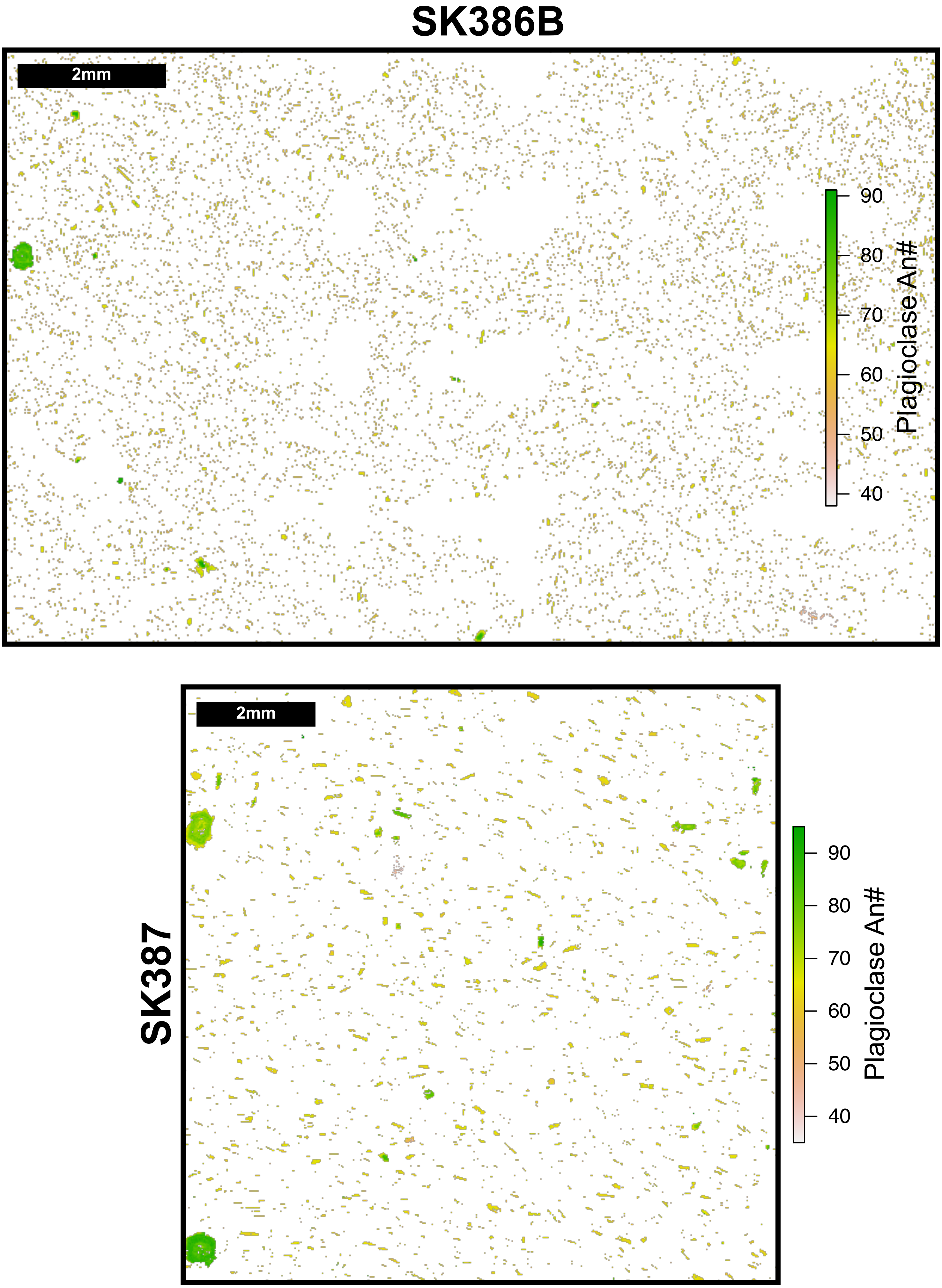
SK386B
2mm
90
80
70
60
50
40
Plagioclase An#
SK387
2mm
90
80
70
60
50
40
Plagioclase An#

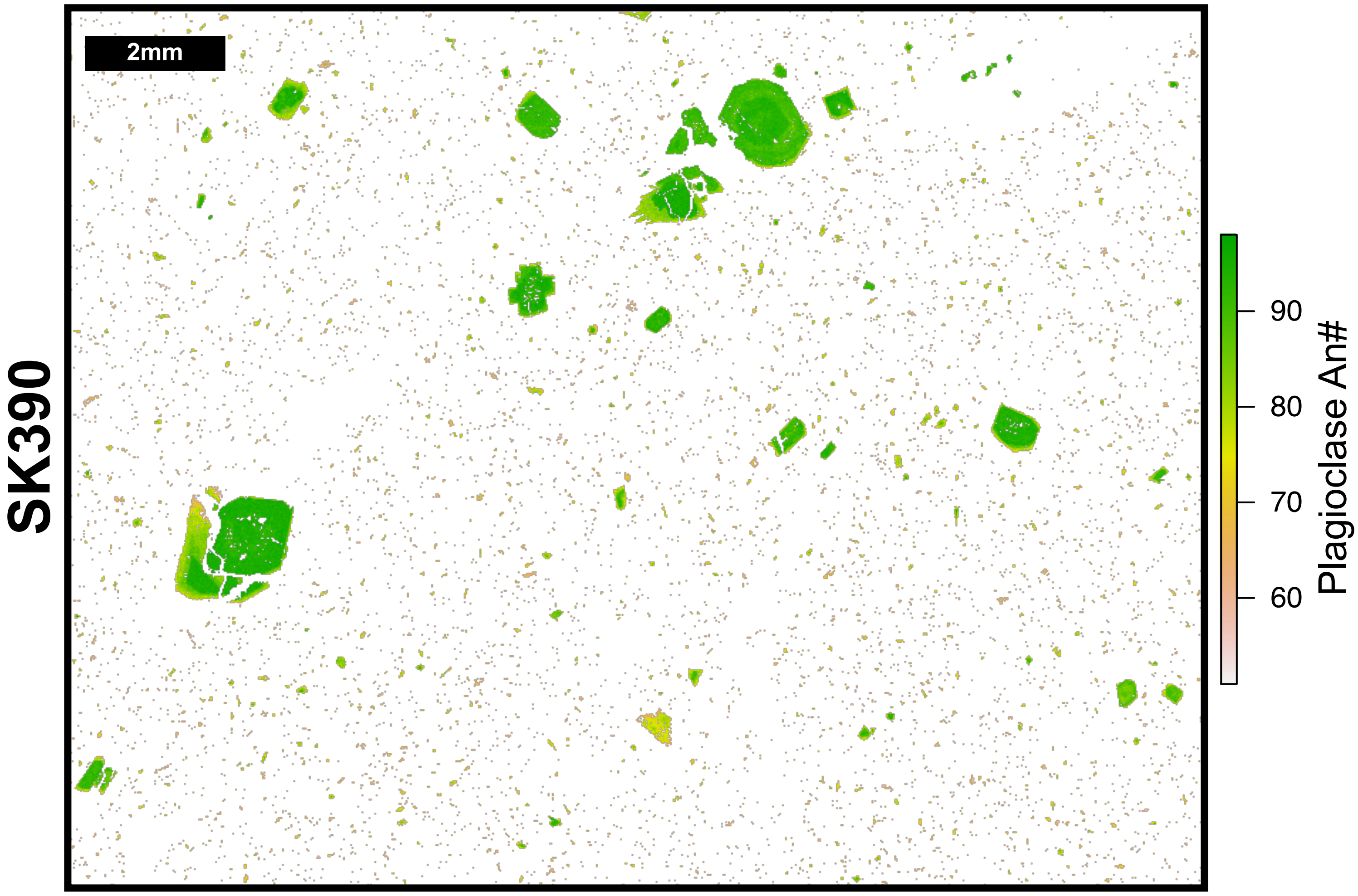
SK390
2mm
90
80
70
60
Plagioclase An#

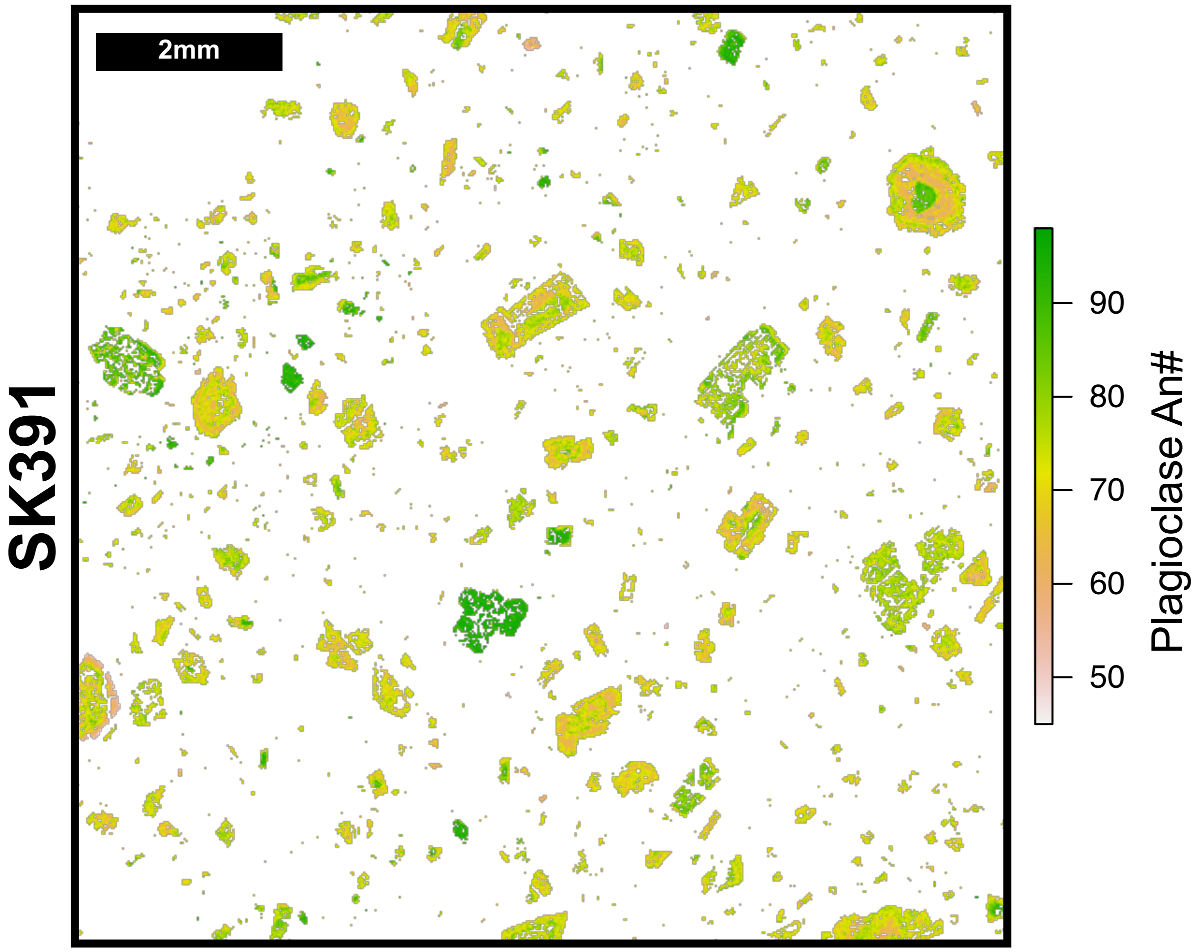
SK391
2mm
90
80
70
60
50
Plagioclase An#

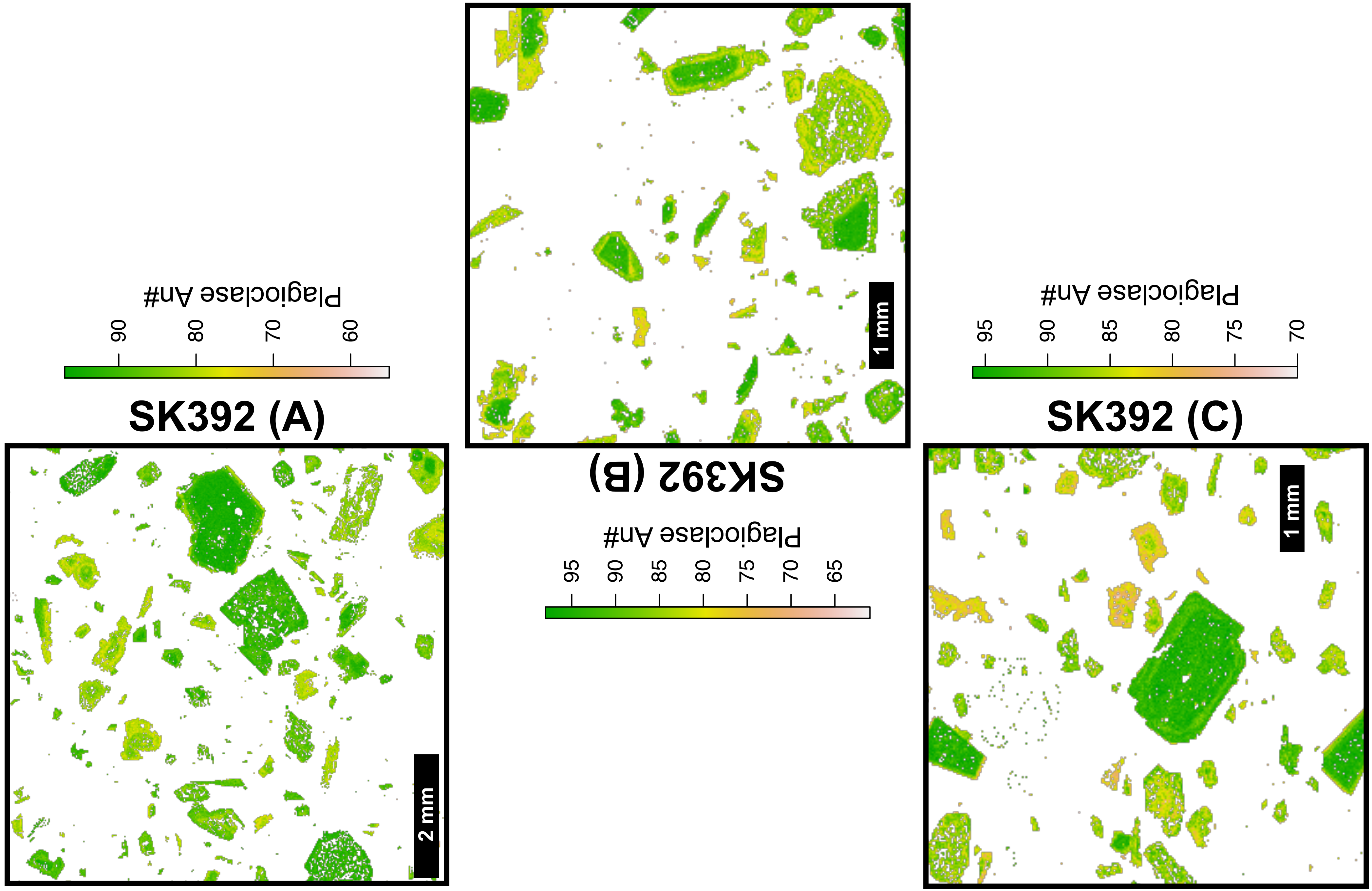
SK392 (A)
Plagioclase An#
90
80
70
60
2 mm
SK392 (B)
Plagioclase An#
95
90
85
80
75
70
65
1 mm
SK392 (C)
Plagioclase An#
95
90
85
80
75
70
1 mm

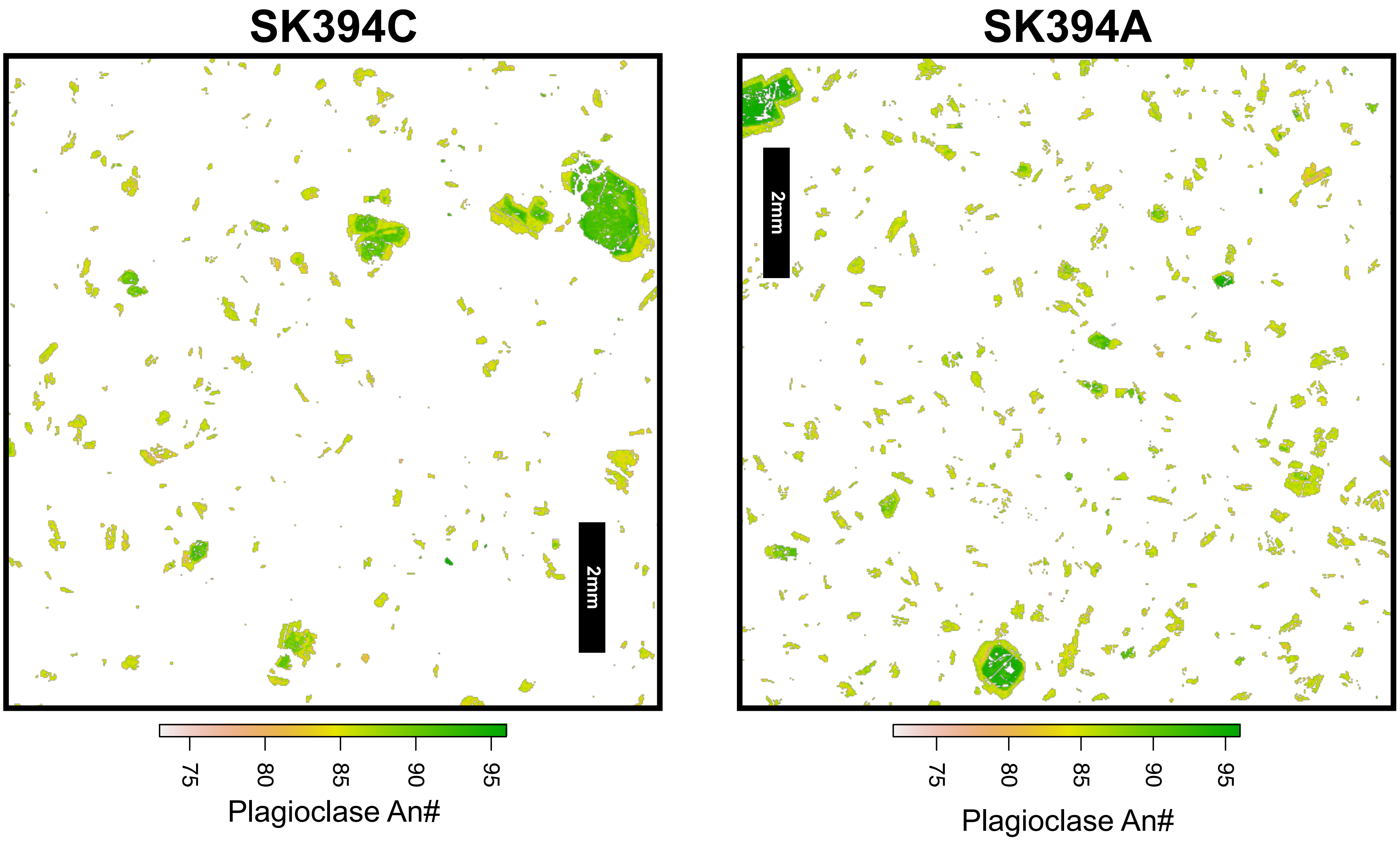

SK394C
2mm
75
80
85
90
95
Plagioclase An#
SK394A
2mm
75
80
85
90
95
Plagioclase An#
