# Supplementary material for: Quantitative chemical mapping of plagioclase as a tool for the interpretation of volcanic stratigraphy: an example from Saint Kitts, Lesser Antilles
Source: Bull Volcanol. 2021 Jul 16;83(8):51. doi: 10.1007/s00445-021-01476-x (PMC8549933; doi:10.1007/s00445-021-01476-x)

Quantitative chemical mapping of plagioclase as a tool for the interpretation of volcanic stratigraphy: an example from Saint Kitts, Lesser Antilles

Bulletin of Volcanology **(Online Resource 7)**

Oliver Higgins*, Tom Sheldrake, Luca Caricchi
Department of Earth Sciences, University of Geneva, rue des Maraîchers 13, 1205, Geneva, Switzerland
*Corresponding author (oliver.higgins@unige.ch; ORCID iD: 0000-0001-9960-934X)

Fig. S4 Zoning groups of plagioclase phenocrysts derived using the textural-chemical image segmentation algorithm of Sheldrake and Higgins (2021)

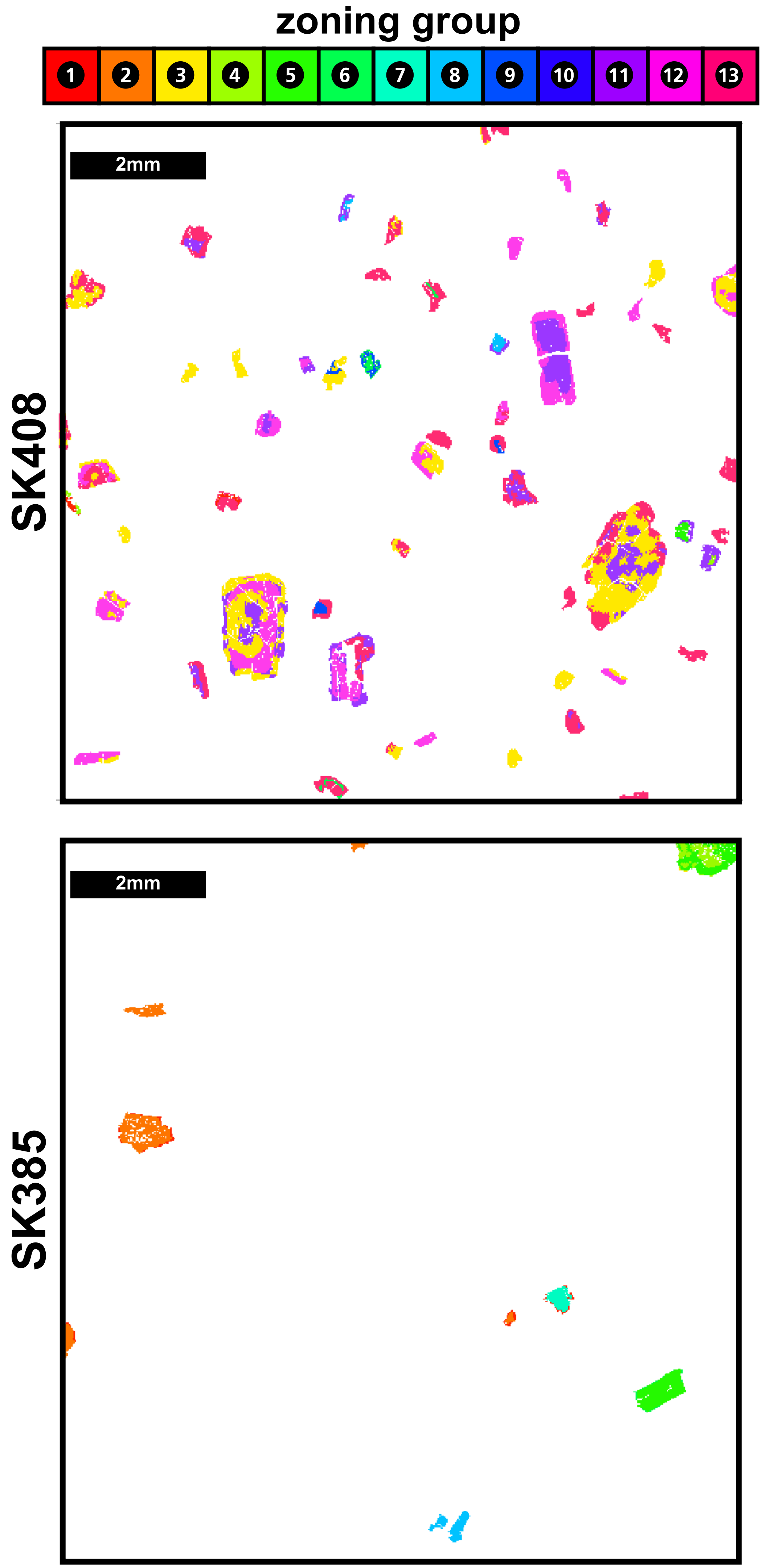
zoning group
1
2
3
4
5
6
7
8
9
10
11
12
13
SK408
2mm
SK385
2mm

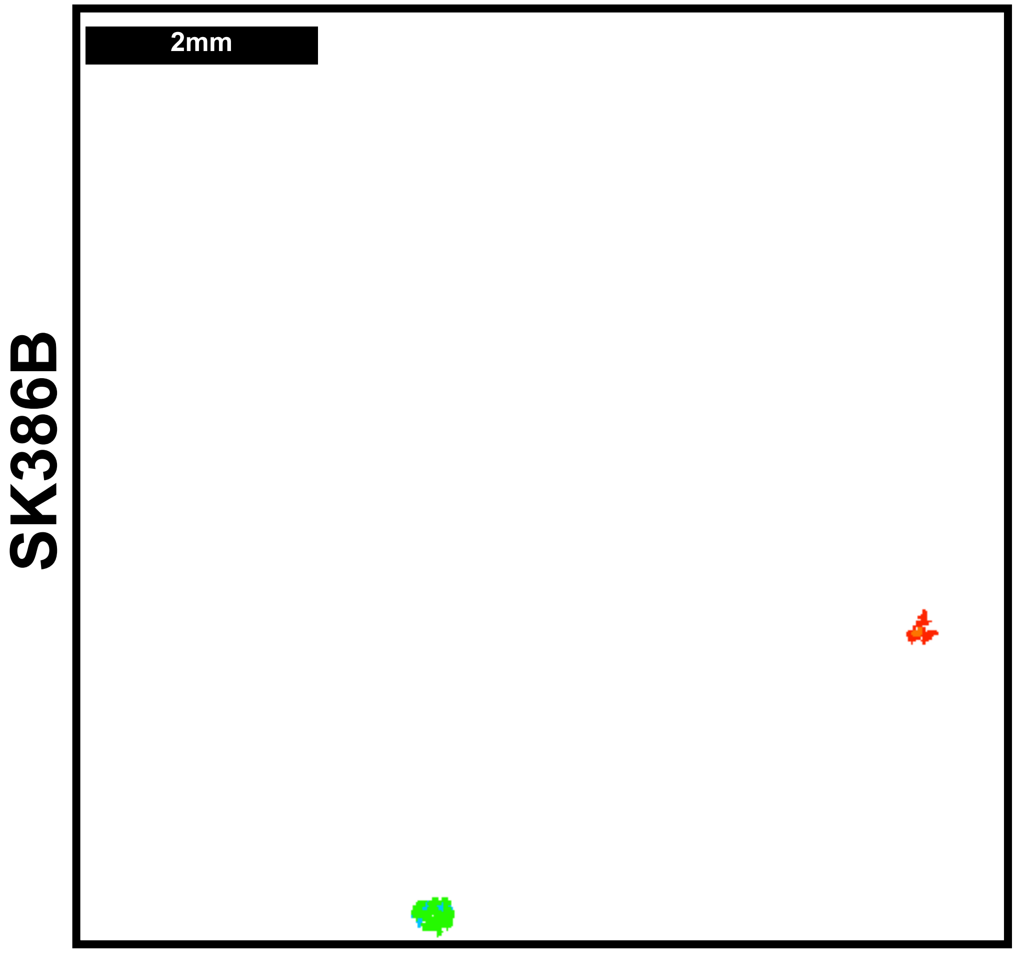
SK386B
2mm

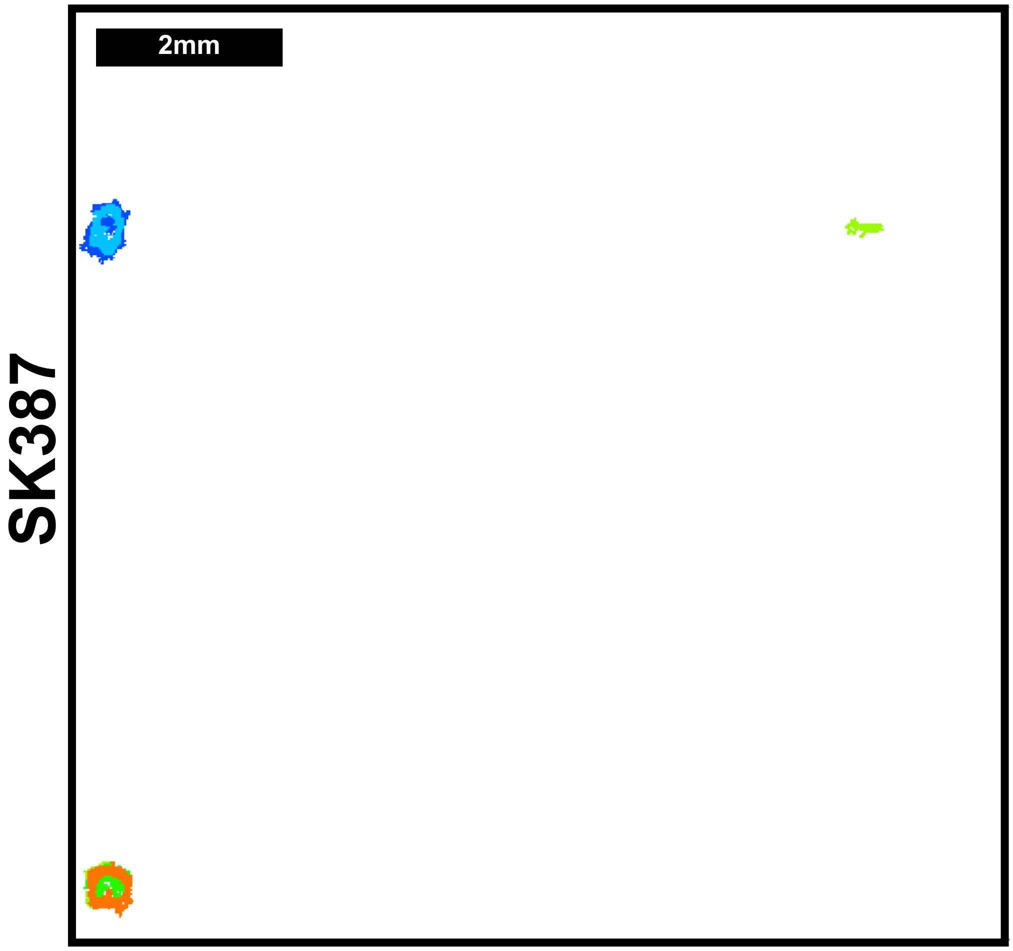
SK387
2mm

SK390

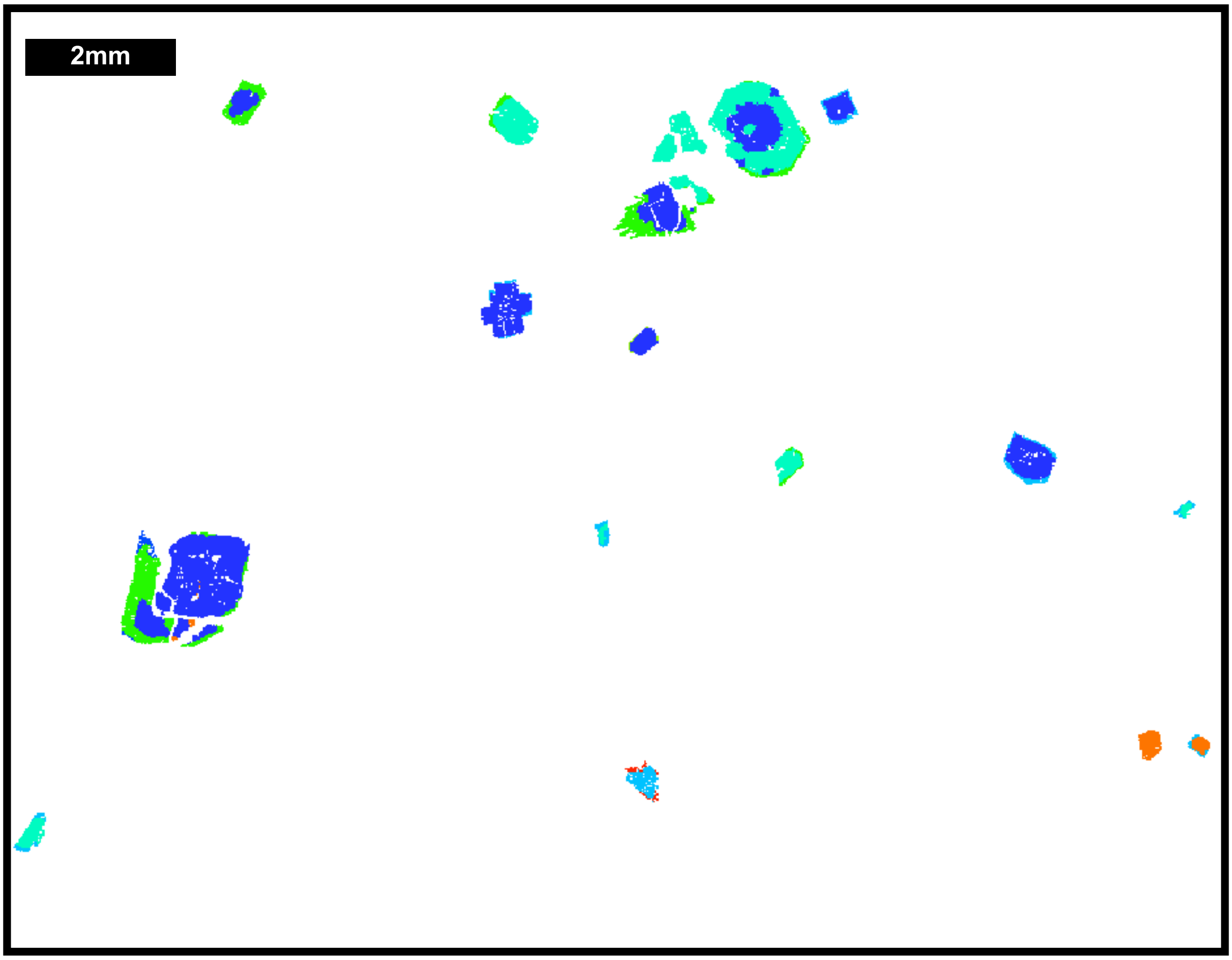

SK391

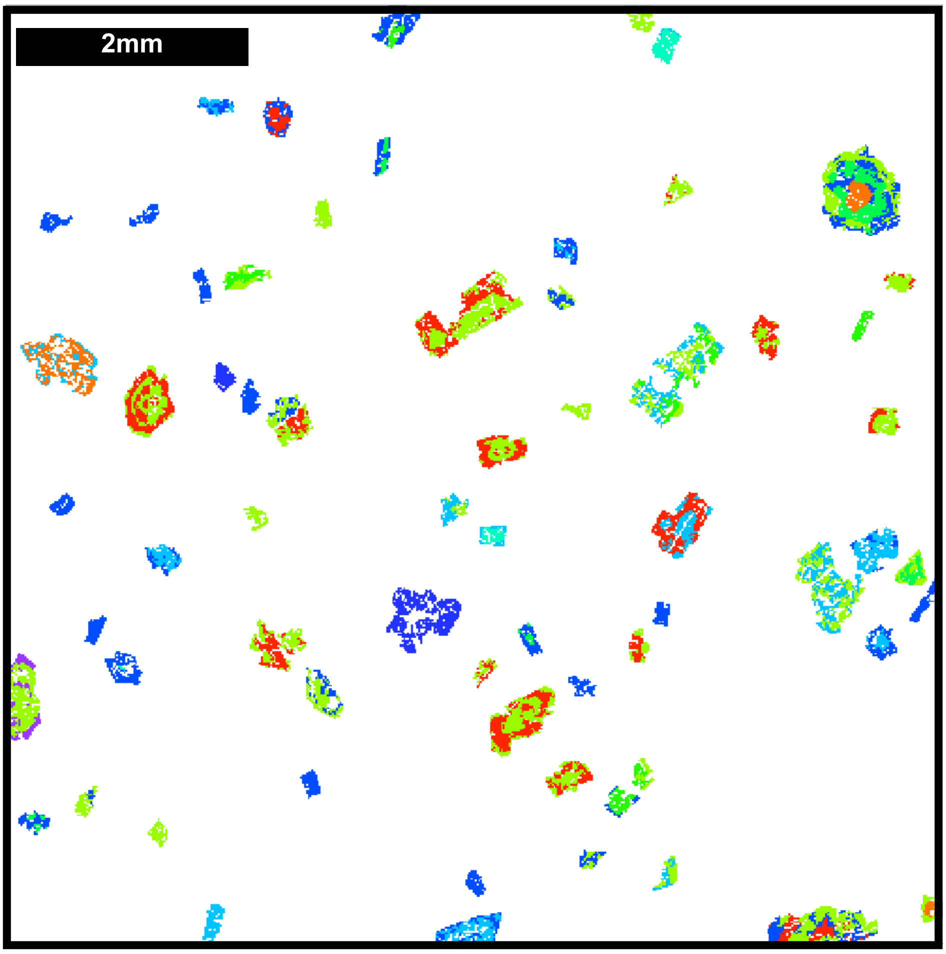

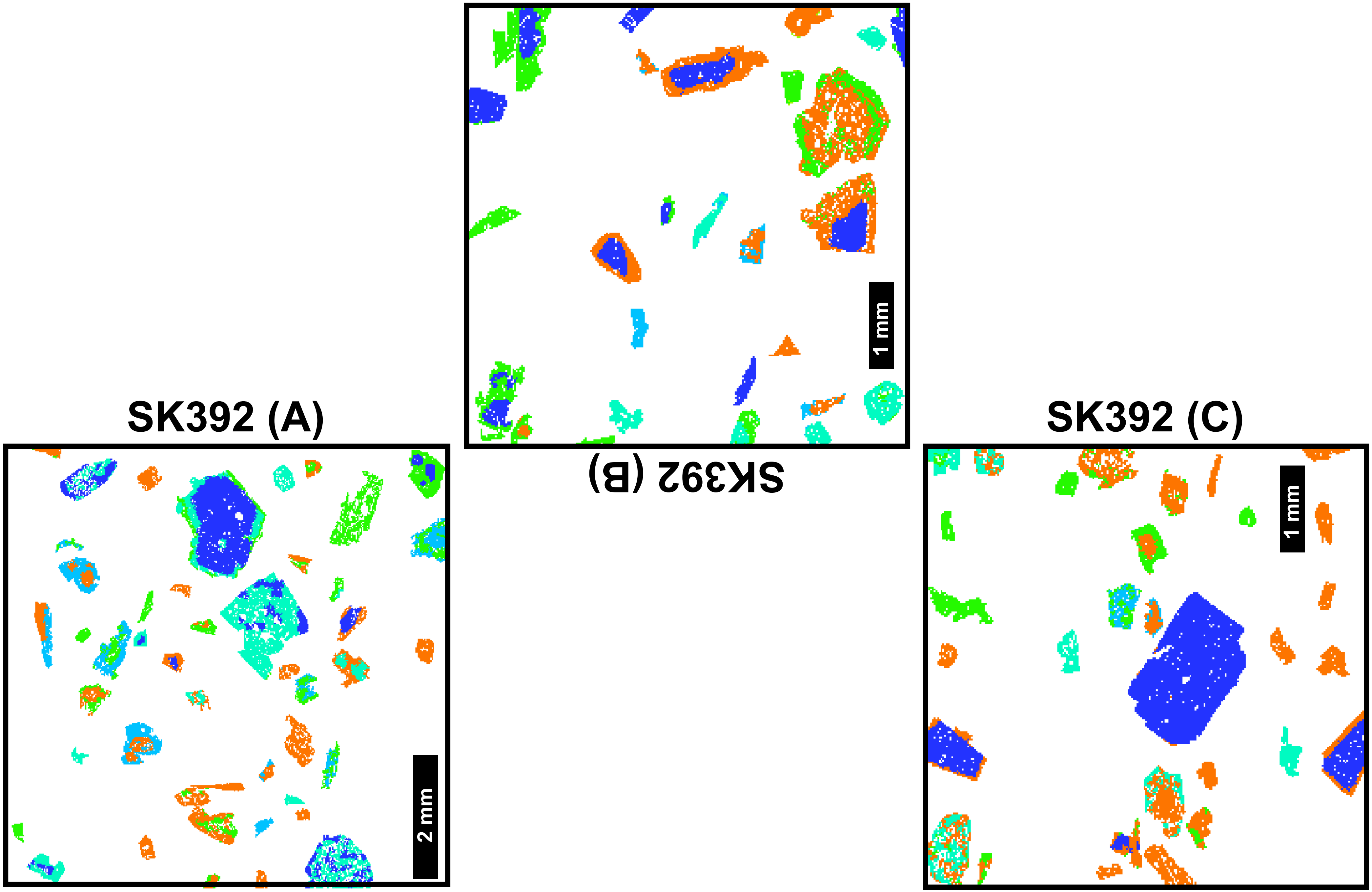
SK392 (A)
2 mm
SK392 (B)
1 mm
SK392 (C)
1 mm

SK394A

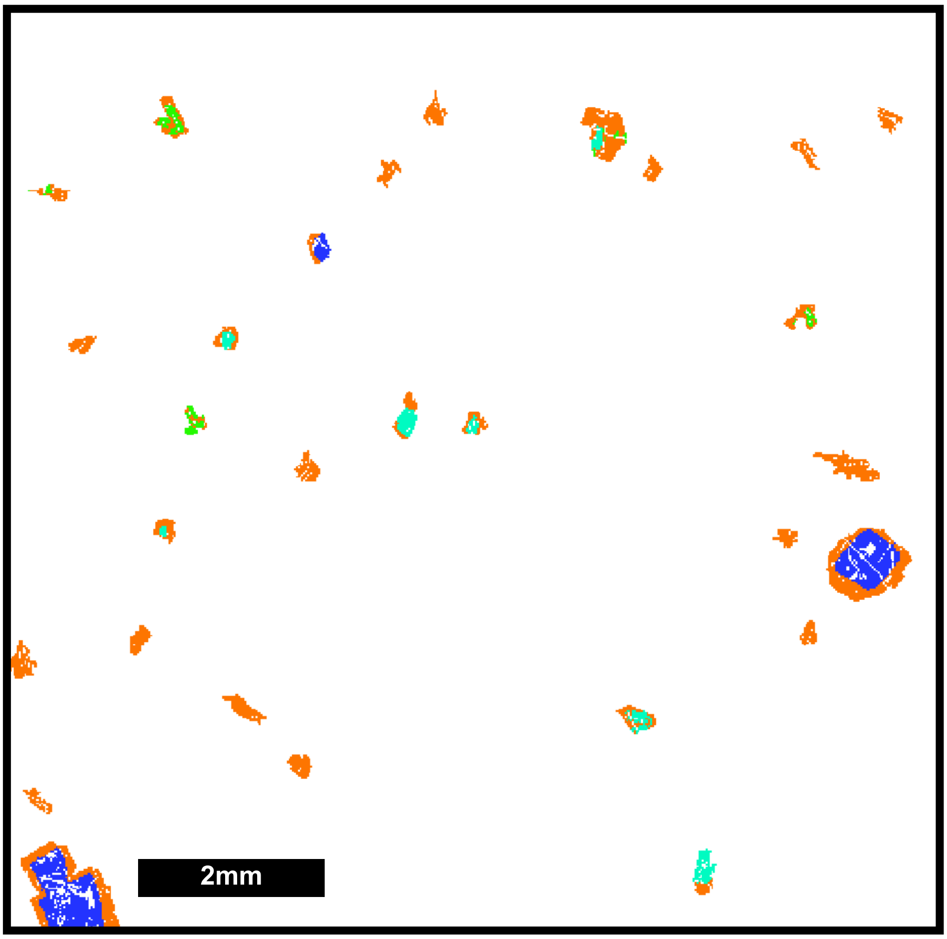

SK394C

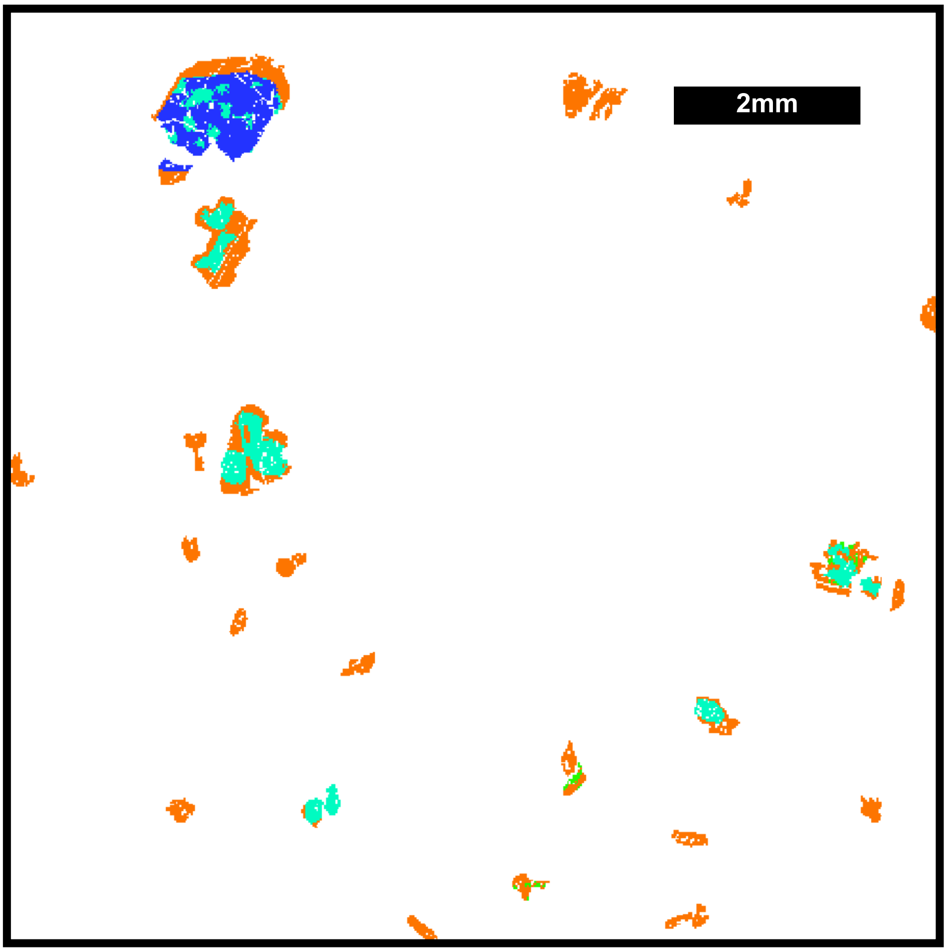

Supplement: Supplementary file 7 — Supplementary file7 (PDF 2478 KB) [file 445_2021_1476_MOESM7_ESM.pdf]
