# Supplementary material for: Quantitative chemical mapping of plagioclase as a tool for the interpretation of volcanic stratigraphy: an example from Saint Kitts, Lesser Antilles
Source: Bull Volcanol. 2021 Jul 16;83(8):51. doi: 10.1007/s00445-021-01476-x (PMC8549933; doi:10.1007/s00445-021-01476-x)
Supplement: Supplementary file 8 — Supplementary file8 (PDF 3950 KB) [file 445_2021_1476_MOESM8_ESM.pdf]

Quantitative chemical mapping of plagioclase as a tool for the interpretation of volcanic stratigraphy: an example from Saint Kitts, Lesser Antilles

Bulletin of Volcanology **(Online Resource 8)**

Oliver Higgins*, Tom Sheldrake, Luca Caricchi
Department of Earth Sciences, University of Geneva, rue des Maraîchers 13, 1205, Geneva, Switzerland
*Corresponding author (oliver.higgins@unige.ch; ORCID iD: 0000-0001-9960-934X)

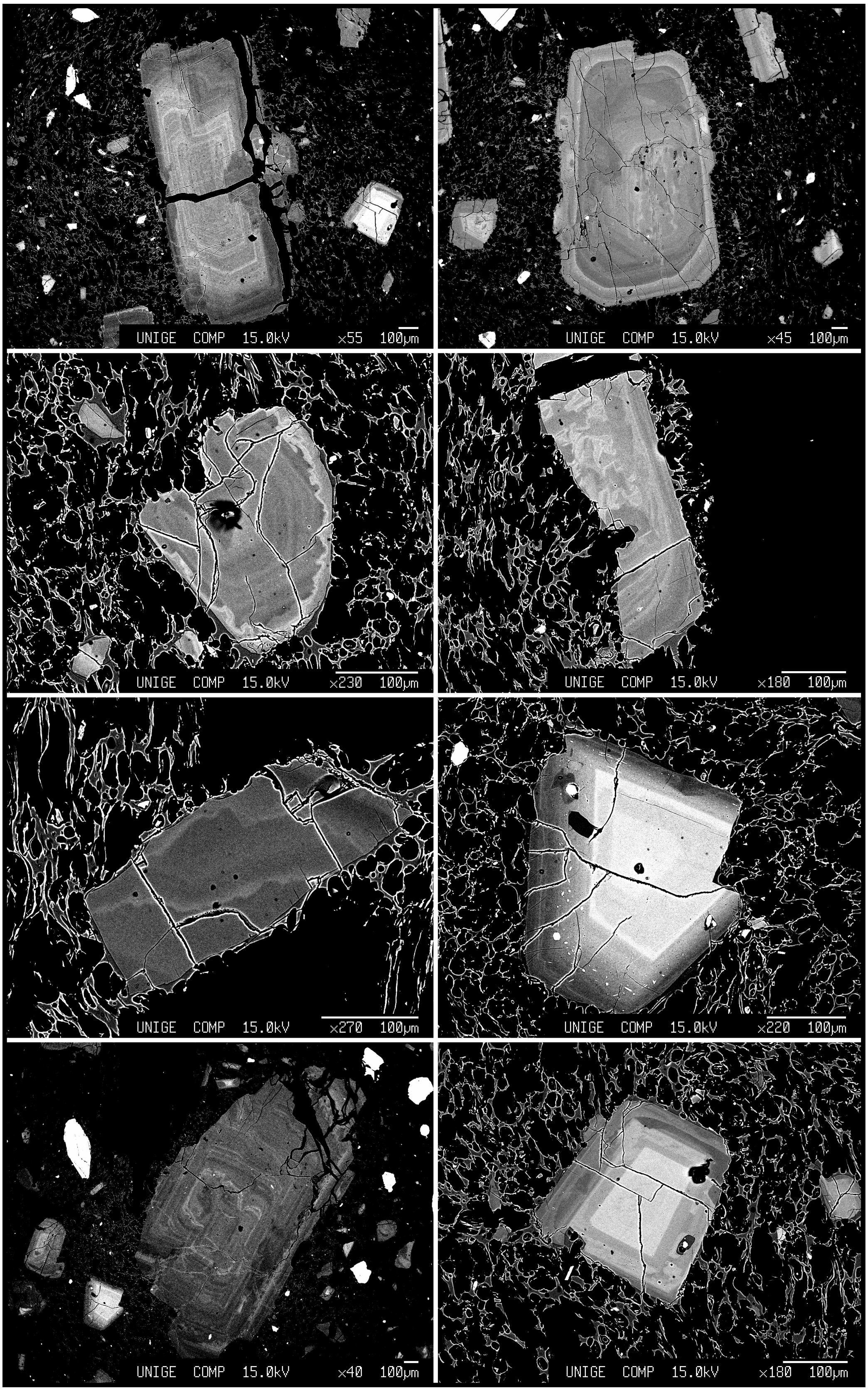

Fig. S5 Exemplar back-scattered electron (BSE) images from sample SK408 used as part of the validation process for the segmentation results
